# Vimar/RAP1GDS1 promotes acceleration of brain aging after flies and mice reach middle age

**DOI:** 10.1038/s42003-023-04822-1

**Published:** 2023-04-15

**Authors:** Ying Xiong, Qi Cheng, Yajie Li, Yanping Han, Xin Sun, Lei Liu

**Affiliations:** 1 Department of Biochemistry and Molecular Biology School of Basic Medicine, Capital Medical University, Youanmen, Beijing, 100069 China; 2 School of Pharmaceutical Science, Jilin Medical University, Jilin City, 132013 China

**Keywords:** Neural ageing, Molecular neuroscience

## Abstract

Brain aging may accelerate after rodents reach middle age. However, the endogenous mediator that promotes this acceleration is unknown. We predict that the mediator may be expressed after an organism reaches middle age and dysregulates mitochondrial function. In the neurons of wild-type *Drosophila* (flies), we observed that mitochondria were fragmented in aged flies, and this fragmentation was associated with mitochondrial calcium overload. In a previous study, we found that mitochondrial fragmentation induced by calcium overload was reversed by the loss of *Vimar*, which forms a complex with Miro. Interestingly, *Vimar* expression was increased after the flies reached middle age. Overexpression of *Vimar* in neurons resulted in premature aging and mitochondrial calcium overload. In contrast, downregulation of *Vimar* in flies older than middle age promoted healthy aging. As the mouse homolog of Vimar, *RAP1GDS1* expression was found to be increased after mice reached middle age; *RAP1GDS1*-transgenic and *RAP1GDS1*-knockdown mice displayed similar responses to flies with overexpressed and reduced *Vimar* expression, respectively. This research provides genetic evidence of a conserved endogenous mediator that promotes accelerated brain aging.

## Abstract (web summary)

Often in the process of aging it is seen that genes and proteins providing normal physiological function at younger ages are detrimental in older age. VIMAR/RAP1GDS1 expression is one such factor with age-dependent functional duality in fly and mouse models.

## Introduction

Brain aging is the most important risk factor for many late-onset neurodegenerative disorders. Whether brain aging follows a gradual decline or an accelerated decline in aged individuals is unclear. Some longitudinal studies in people aged 20–90 suggested that the cognitive function decline in each decade follows a linear trend, indicating that a single factor mediates brain aging^1^. Other studies demonstrated that brain atrophy precedes cognitive decline and is accelerated in advanced age; this neuroanatomical change was used to predict brain pathology development^2,3^. In addition, different brain regions exhibited distinct degrees of brain volume loss during aging^4^.

Although the molecular mechanisms underlying accelerated brain aging are unknown, many factors have been reported to contribute to the deterioration in the brain during aging, such as the accumulation of mutations in mitochondrial DNA and genomic DNA, increased oxidative stress induced by mitochondrial dysfunction, enriched pathological proteins, decreased protein degradation pathway activity, elevated inflammatory cytokine levels, a reduction in trophic factor levels, altered epigenetic regulation, and metabolic dysfunction^3,5,6^. Among these factors, the dysfunction of mitochondrial metabolism clearly plays a prominent role in the development of brain disorders^7,8^. Consistent with this widely accepted cause of brain disease, the rate of daily total energy expenditure remains constant before an organism reaches middle age but significantly declines after middle age, at ~60–63 years in people^9^. Closely associated with the metabolic (mitochondrial) theory of aging, a subtle and gradual increase in cytosolic calcium levels has been proposed to contribute to brain aging, and the loss of calcium homeostasis may be induced through multiple mechanisms, including increased calcium influx through voltage-gated channels, calcium accumulation of intracellular organelles, changes in the rate of calcium release and altered calcium-buffering systems^10^. Despite this mechanistic understanding, the identity of an endogenous mediator that promotes accelerated brain aging is less clear.

In a previous study, we identified *Drosophila* Vimar as the guanine nucleotide exchange factor (GEF) associated with Miro, a small GTPase known to regulate mitochondrial morphology and molecular transport^11^. We demonstrated that the Miro/Vimar complex played a key role in mitochondrial fragmentation induced by intracellular calcium overload. RAP1GDS1 is the mouse homolog of Vimar, which functions as a GEF for several small GTPases, including RHOA, RAC1, KRAS and Miro1, and RAP1GDS1 regulates cell migration and proliferation mediated through DNA synthesis, molecular chaperone functions and protein posttranslational modifications^11,12^.

In this study, we showed that *Vimar/RAP1GDS1* expression was increased in neurons in the brain in organisms older than middle age. This alternation may enhance Miro function in mitochondria and induce mitochondrial calcium overload and fragmentation. This study indicates that Vimar/RAP1GDS1 is an endogenous mediator that accelerates brain aging in flies and mice that have lived past middle age.

## Results

### Accelerated brain aging in wild-type *Drosophila*

**Fig. 1:**
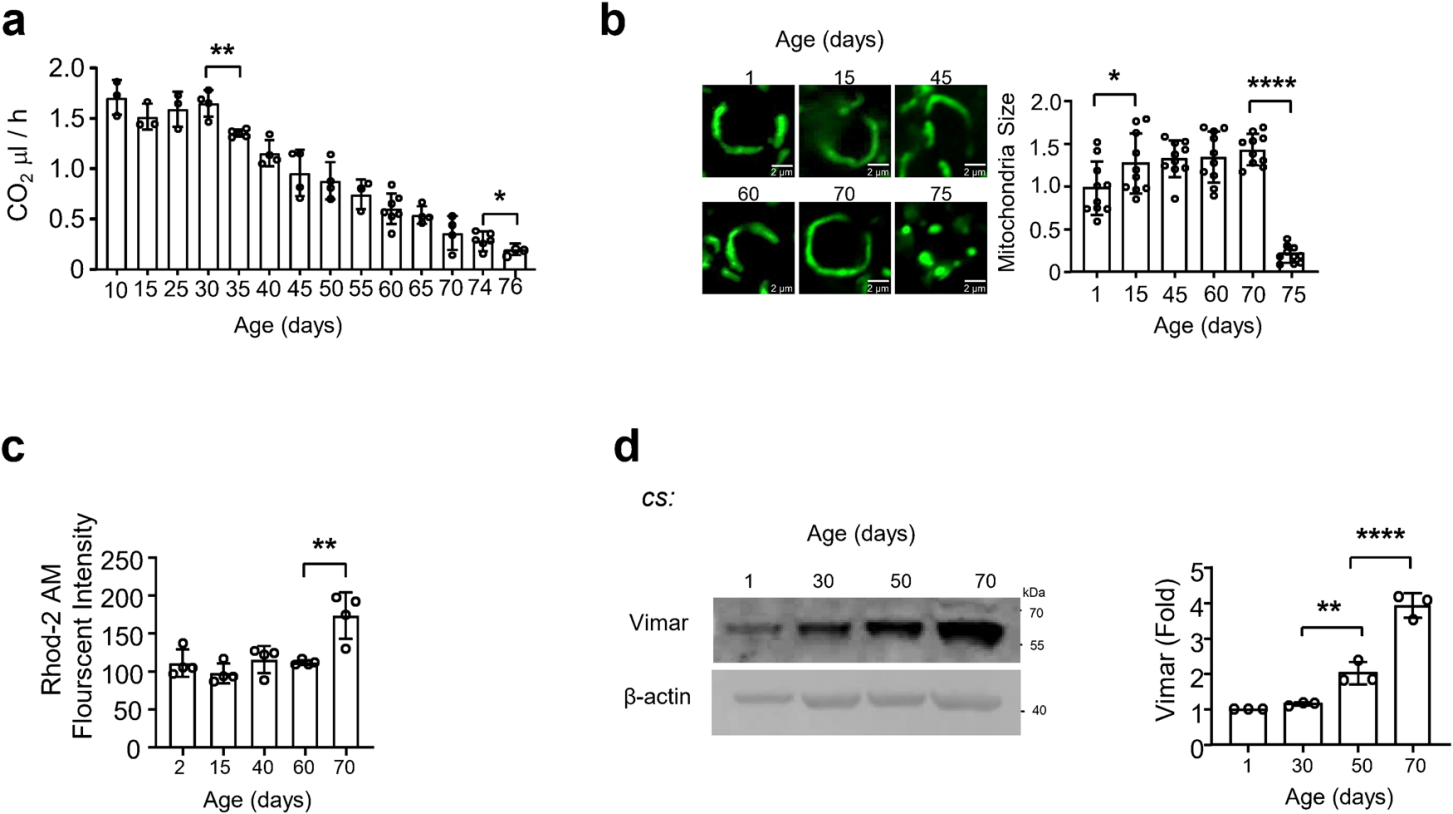
Characterization of mitochondria-related functions in the life course of wild type flies. **a** The daily energy expenditure measured by CO2 production at different ages of the wild type CS adult flies. 15 flies were tested for each experiment. Trial *N* = 3–7. One-way ANOVA with Tukey’s post hoc test. **P = 0.0008, *P = 0.0204. **b** Representative mitochondrial morphology in the brain around mushroom body. All mitochondrial images are Z-stacked throughout all figures. Mitochondria are labeled with *UAS-mitoGFP*, which is driven by *Appl-Gal4* (*Appl* > *mitoGFP*). The age is indicated on each graph. Scale bars, 2 μm. For all mitochondrial size and length quantification throughout all figures, individual mitochondrion was identified by ImageJ from a 3-D image convolution in a single neuron, with 10 neurons measured in each sample. The averaged mitochondrial size from 1 day-old is set as 1, and the relative ratios of the other ages are shown. Three area were averaged for each brain sample. Trial *N* = 10. One-way ANOVA with Tukey’s post hoc test. *P = 0.0136, ****P < 0.0001. **c** Mitochondrial calcium concentration ([Ca^2+^] _mito._) change during aging. The intensity of Rhod2-AM indicates the relative level of [Ca^2+^]_mito_. Trial *N* = 4. One-way ANOVA with Tukey’s post hoc test. **P = 0.0026. **d** Representative micrograph of Vimar protein level during aging. β-actin is shown as the protein loading control from the same blot. Uncropped blots are shown in Supplementary Fig. 7. The band intensity of 1 day-old brain sample is set as 1, and the relative ratios of the other ages are shown. Trial *N* = 3. Error bars are mean ± SE. One-way ANOVA with Tukey’s post hoc test. **P = 0.0091, ****P < 0.0001.

To determine whether brain aging in *Drosophila* follows a linear or accelerated decline in the course of life, we examined energy expenditure, which is a key parameter of mitochondrial function and plays crucial roles in brain aging^9^. Under our culture conditions, wild-type *Canton Special* (*CS*) flies survived as long as 82 days. Using a CO_2_ consumption method^13^, we found that CO_2_ production significantly declined after the flies reached 30, 65 and 74 days of age (Fig. 1a). Moreover, the brain ATP level significantly declined after they reached 35 and 50 days of age (Supplementary Fig. 1a). These results indicated that mitochondrial metabolism significantly declined in flies who lived past middle age (30 days old) and markedly declines in flies of advanced age (75 days old), which were findings consistent with studies of mammalian brains showing that mitochondrial respiration continuously declined during aging^7^. Mitochondrial number can be quantified on the basis of the mitochondrial DNA (mtDNA) content^14^. Consistent with the decreased energy output during aging, the mtDNA content in the brain declined in flies of advanced age (70 days old) (Supplementary Fig. 1b). To assess morphological changes in the mitochondria of neurons, a neuron-specific promoter (*Appl-Gal4*) was used to drive *UAS-mito GFP*, a marker of mitochondria, in vivo. Compared with the mitochondria in the 1-day-old fly brain, the mitochondria were enlarged in the 15-day-old fly brain, and this increasing trend was maintained until the flies reached 70 days of age. Notably, the mitochondria became fragmented in mice who lived 75 days (Fig. 1b). These results indicated that mitochondrial function may gradually decline in flies from 30 to 70 days old and reach a stage of severe dysfunction when the flies are ~75 days old. Because mitochondrial fragmentation and damage are commonly associated with neurodegenerative conditions^15^, 75 days may represent a late stage in fly brain aging.

To study the mechanism of mitochondrial fragmentation in aged flies, we measured cytosolic and mitochondrial calcium levels. Calcium plays a key role in the regulation of mitochondrial morphology and metabolism, and calcium overload is commonly found in aged neurons^16,17^. Using Fura-2 AM to measure cytosolic calcium levels^18^ and Rhod-2AM to measure mitochondrial calcium levels^19^, we observed that [Ca^2+^]_cyto._ was elevated in flies that reached 60 days old and the [Ca^2+^]_mito._ was significantly elevated in the late stage of aging (70 days old) (Fig. 1c and Supplementary Fig. 1c). Mitochondrial calcium overload was identified a few days before mitochondrial fragmentation was observed. This observation was consistent with reports by others suggesting that mitochondrial calcium overload induced mitochondrial fragmentation^20^. Similarly, our previous study suggested that calcium overload in *Drosophila* neurons induced mitochondrial fragmentation and cell death mediated through the Vimar/Miro and Drp1 pathways, and loss-of-function *Vimar*, a guanine exchange factor (GEF) associated with Miro, rescued mitochondrial fragmentation without reducing the calcium overload^11^. These results inspired us to examine alterations in the Vimar protein during aging. Notably, the Vimar protein level gradually increased after flies reached middle age (30 days old) and was further elevated in both wild-type lines *CS* and *w*^*1118*^ flies that reached 50 days old (Fig. 1d and Supplementary Fig. 1d). In wild-type flies (*CS* and *w*^*1118*^ flies), the transcription of *Vimar*, *Miro*, and *DRP1* was increased at the 50 day mark; *OPA1*, *MFN1*, and *MFN2* levels were unaltered (Supplementary Fig. 1e, f). Similarly, neuron-specific overexpression of *Vimar* led to increased *Drp1* mRNA levels (Supplementary Fig. S1g), and *Vimar* RNAi suppressed this increase in *Drp1* mRNA levels (Supplementary Fig. S1h). This finding suggests that Vimar may somehow regulate the expression of *Drp1*. It also indicates that mitochondrial fragmentation in the late aging stage may be regulated by Vimar, Miro and DRP1.

### Upregulation of the Miro/Vimar complex is the turning point in the accelerated brain aging in *Drosophila*

**Fig. 2:**
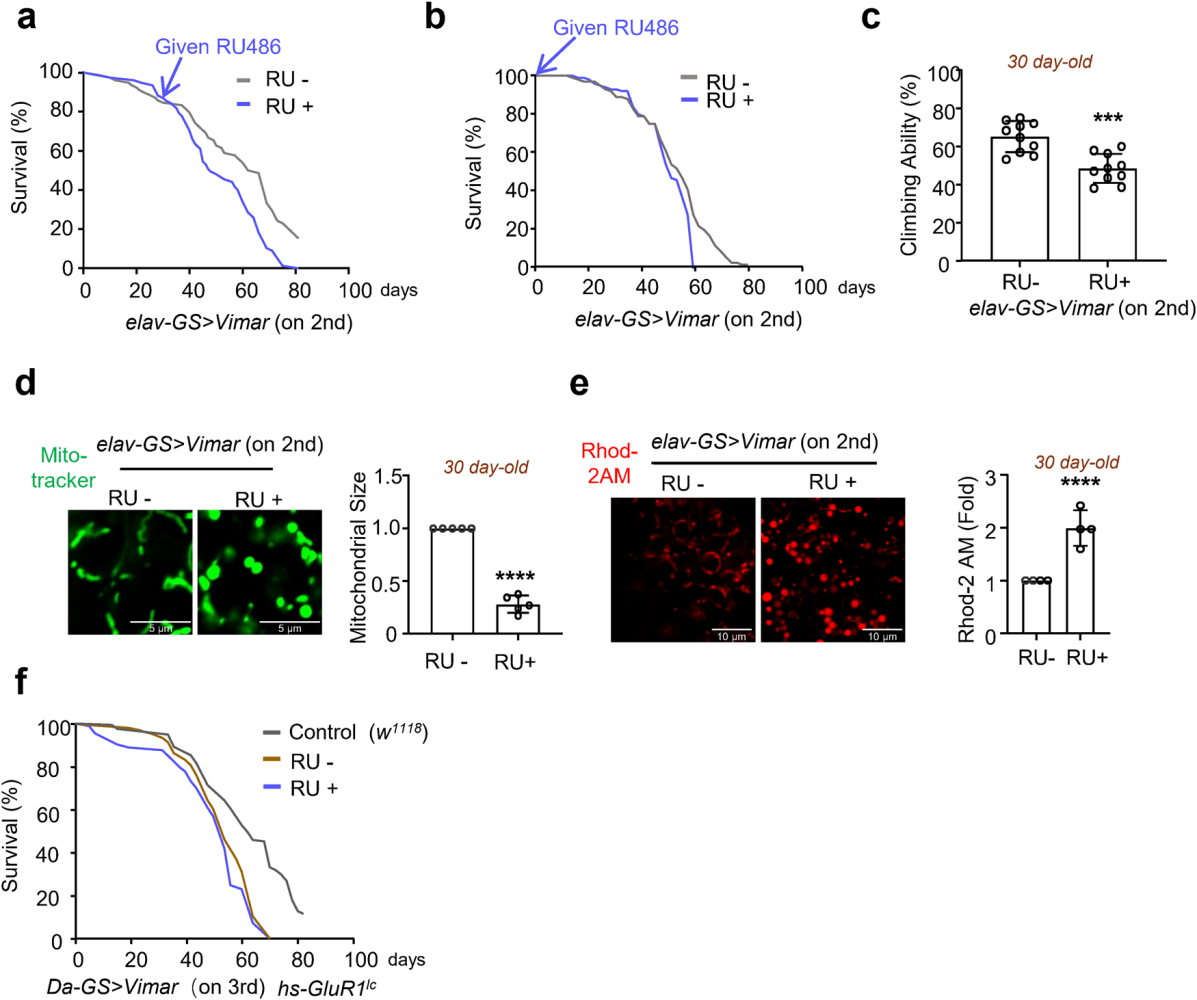
Characterization of Vimar in brain aging. **a** Survival curve of *Vimar* overexpression. Neuron-specific gene-switch Gal4 (*elav-GS-Gal4*) was used to dive *UAS-Vimar* expression, starting from 30 day-old by feeding flies with RU486 (RU+). Each vial contained 10 females and 10 males adult flies. Total flies tested, *N* = 166 for RU−, *N* = 177 for RU+. Median lifespan: RU− = 67 days, RU+ = 53 days. Long-rank test, *P* = 0.0018. **b** The same condition as in **a**. With RU fed from the 1 day-old flies. Total flies tested, *N* = 180 for RU−, *N* = 165 for RU+. Median lifespan: RU− = 54 days, RU+ = 48 days. Long-rank test, *P* = 0.0005. **c** The climbing ability assay. With RU fed from the 1 day-old *elav-GS* > *Vimar* flies. 30 days old flies were tested, with 20 flies tested for each trial. Trial *N* = 7. Unpaired t-test. ***P < 0.0001. **d** Representative neuronal mitochondrial morphology of live imaging by Mito Tracker green staining. The genotype is *Elav-GS-Gal4* > *Vimar*. The flies were 30 day-old, scale bar, 5 μm. The average mitochondrial size of control (RU−) is set as 1, and the relative ratios of RU+ are shown. Trial *N* = 6. Unpaired t-test. ****P < 0.0001. **e** Representative live images of mitochondrial calcium measurement by Rhod2-AM. The genotype is *elav-GS-Gal4* > *Vimar*. The flies were 30 day-old, scale bar, 10 μm. The average intensity of Rhod 2-AM of control (RU−) is set as 1, and the relative ratio of RU+ are shown. Three area (400 μm^2^) were averaged for each brain sample. Trial *N* = 4. Error bars are mean ± SE. Unpaired t-test. ****P < 0.0001. **f** Survival curve of *Vimar* overexpression under the calcium overload background. The genotype is wild type control (*w*^*1118*^), *elav-GS* > *Vimar; hs-GluR1*^*Lc*^ (RU−) and (RU+). With 10 female and 10 male flies in each vial. The total tested flies, *N* = 139 (*w*^*1118*^), *N* = 166 (RU−), and *N* = 177 (RU+). Median lifespan: *w*^*1118*^ = 61 days, RU− = 54 days, RU+ = 53 days. Long-rank test, *P* = 0.0028 (*w*^*1118*^ vs RU+); *P* = 0.0024 (*w*^*1118*^ vs RU−).

Next, we assessed whether Vimar overexpression induces premature aging. GeneSwitch (GS) is a modified Gal4/UAS system that drives transgene expression after the drug RU486 is added to fly food. The gene switch system can activate gene expression after the developmental stage^21^. Using the promotors *DA (daughterless)-GS-Gal4* and *elav-GS-GAL4* to drive *UAS-Vimar* expression specifically in neurons (*elav-GS* > *Vimar*) or ubiquitously in many cell types (*DA-GS* > *Vimar*), *Vimar* expression was initiated at different adult stages, namely at 1 day or 30 days old, by feeding flies RU486 (mifepristone). The control flies were of the same genotype and were not fed RU486. RU486 (500 μM) fed to wild-type *Drosophila* (*DA-GS* > *GFP*) did not affect the lifespan (Supplementary Fig. 2a), consistent with reports by others^22^. We generated two independent lines with insertion of *UAS-Vimar* into the 2nd or 3rd chromosome. A lifespan assay demonstrated that the expression of *Vimar* (*elav-GS* > *Vimar* (in the 2nd chromosome)) from Day 30 or Day 1 was associated with a shortened lifespan (Fig. 2a, b; and Supplementary Fig. 2b). Similarly, *DA-GS-Gal4-*driven *UAS-Vimar* (*DA-GS* > *Vimar* (in the 3rd chromosome)) showed a shorter lifespan (Supplementary Fig. 2c, d). A climbing assay can be performed to assess aging^23^. Compared to control flies, both *elav-GS* > *Vimar* (in the 2nd chromosome) and *DA-GS* > *Vimar* (in the 3rd chromosome flies showed premature climbing deficits (Fig. 2c and Supplementary Fig. 2e). It appears that the climbing defect conferred by *elav-GS* > *Vimar* presented earlier than the *DA-GS* > *Vimar* defect, likely due to the different promoters (*elav-GS* vs. *DA-GS*) and Vimar insertion sites (the 2nd vs. the 3rd chromosome). Because both *Vimar*-transgenic lines showed behavioral defects after expression, the insertion sites were unlikely to be a contributing factor. MitoTracker Green was used to assess mitochondrial morphology. Compared with the mitochondria in the 30-day-old control flies (*elav-GS* > *Vimar* flies not fed RU486), the mitochondria in the *elav-GS* > *Vimar* flies (fed RU486) were swollen and smaller (Fig. 2d). Ubiquitous Vimar overexpression (*DA-GS* > *Vimar* (in the 3rd chromosome)) induced similar mitochondrial swelling (Supplementary Fig. 2f) without inducing apoptosis (Supplementary Fig. 2g). Consistent with premature aging, the *elav-GS* > *Vimar* flies showed reduced daily metabolism rates, brain ATP levels, and citrate synthase (CS) activity, indicator of Krebs cycle activity in mitochondria (Supplementary Fig. 2h–j). After staining with tetramethylrhodamine and methyl ester (TMRM), the mitochondrial membrane potential was found to be lost in Vimar-overexpressing flies (Supplementary Fig. 2k). Moreover, *Vimar* overexpression promoted an [Ca^2+^]_mito_ increase, as assessed by Rhod-2AM assay (Fig. 2e). Together, these results suggest that increased *Vimar* expression after middle age may promote brain aging in *Drosophila*.

The Miro/Vimar complex promotes mitochondrial fragmentation under calcium overload conditions^11^. To test whether *Vimar* overexpression in a calcium overload setting may exert an additive effect on brain aging, we crossed *DA-GS-Gal4* > *Vimar* flies with *hs-GluR1*^*Lc*^ model flies, which showed mild calcium overload, as has been previously reported^24^. We verified that *hs-GluR1*^*Lc*^ induced calcium overload (Supplementary Fig. 2l). Compared to that of the wild-type flies (*DA-GS-Gal4/+*) and flies with calcium overload (*DA-GS-Gal4* > *Vimar*; *hs-GluR1*^*Lc*^ not fed RU486), the lifespan of the *DA-GS-Gal4* > *Vimar* (in the 3rd chromosome); *hs-GluR1*^*Lc*^ (fed RU486) was shorter and similar, respectively (Fig. 2f). Moreover, mitochondrial function-related parameters, such as brain ATP level (at 30 days old), CS activity (at 30 days old) and oxidative stress response (at 10 days old, induced by feeding the flies paraquat), showed no differences in the *Vimar* overexpression and *Vimar* overexpression plus *GluR1*^*Lc*^ expression groups (Supplementary Fig. 2m–o). These findings suggest that *Vimar* overexpression and calcium overload do not exert an additive effect, indicating that they mediated through the same pathway. In support for this conclusion, our previous data showed that calcium overload-induced mitochondrial fragmentation and cell death were rescued by the loss of function of *Vimar*^11^.

**Fig. 3:**
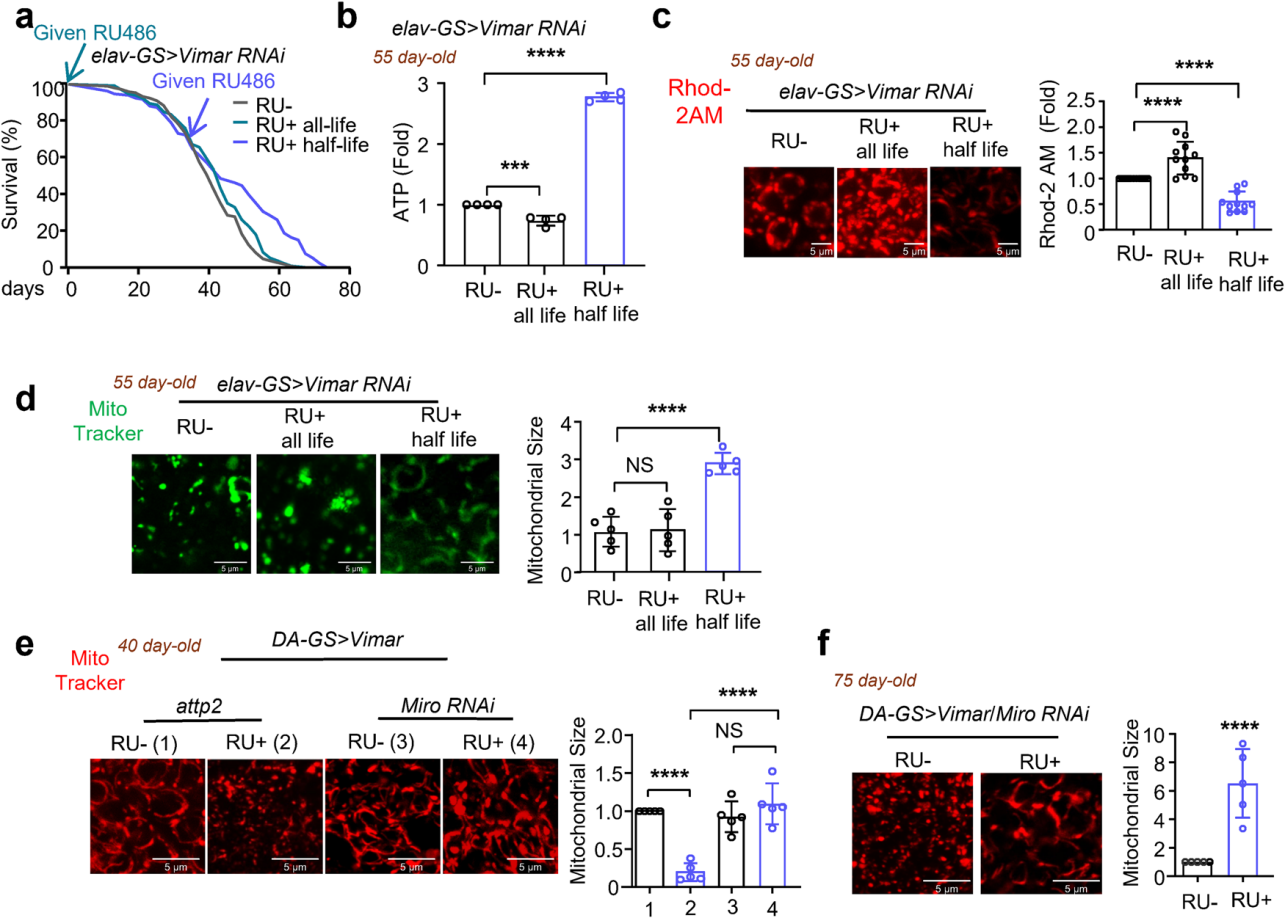
Effect of Vimar RNAi on aging. **a** Survival curve of *elav-GS-Gal4* > *Vimar RNAi* flies. The gray line is the control without induction of *Vimar RNAi* (RU−). The blue line is given RU486 from 1 day-old and kept on whole life (RU+ all-life). The red line is given RU486 from 30 day-old and kept on the rest of life (RU+ half-life). Each vial contained 10 female and 10 male flies. Total flies tested *N* = 179 (RU−), *N* = 134 (RU+ all life), *N* = 134 (RU+ late). Median lifespan: RU− = 48 days, RU+ all-life = 49 days, RU+ half-life = 54 days. Long-rank test, *P* = 0.689 (RU− vs RU+ all-life); *P* = 0.00276 (RU− vs RU+ half-life). **b** Brain ATP level. The same fly group as **a** is tested, with 55 day-old of flies. Each experiment collected 40 fly heads. The average ATP level of control (RU−) was set as 1, with the ATP level was normalized to protein concentration. The relative ratios of the other flies are shown. Trial *N* = 4. One-way ANOVA with Tukey’s post hoc test. ***P = 0.0005, ****P < 0.0001. **c** Representative live image of mitochondrial calcium level measured by Rhod2-AM. The same fly group as **a** is tested, with 55 day-old of flies. Scale bar, 5 μm. The averaged intensity of control (RU-) was set as 1, and the relative ratios of RU+ all-life and RU+ half-life to the control (RU−) are shown. Three area (400 μm^2^) were averaged for each brain sample. Trial *N* = 11. One-way ANOVA with Tukey’s post hoc test. ****P < 0.0001, ****P < 0.0001. **d** Representative live image of mitochondrial morphology. The same fly group as **a** is tested, with 55 day-old of flies. Mitochondria are labeled by Mito Tracker green, scale bar, 5 μm. The bar graph showed the quantification, flies tested *N* = 5. One-way ANOVA with Tukey’s post hoc test. “NS” for not significant, *P* = 0.765, ****P < 0.0001. **e** Representative live image of mitochondrial morphology. Mitochondria are labeled by Mito Tracker red, scale bar, 5 μm. The “attp2” genotype is *DA-GS-Gal4*; *UAS-Vimar/attp2*; and “Miro RNAi” is *DA-GS-Gal4; UAS-Vimar/Miro RNAi*. 40 day-old of flies were tested. The average mitochondrial size of control (RU-) was set as 1, and the relative ratios of other flies are shown. Tested fly *N* = 5. One-way ANOVA with Tukey’s post hoc test. ****P < 0.0001, ****P < 0.0001, “NS” for not significant, *P* = 0.188. **f** Representative live image of mitochondrial morphology. The genotype is *DA-GS-Gal4* > *Vimar/MiroRNAi*, fed with RU486 or not. The control *DA-GS-Gal4* > *Vimar/attp2* (RU−) is the genetic matched background of above fly line. 40 day-old were tested. Mitochondria is labeled by Mito Tracker red. Scale bar, 5 μm. The average mitochondrial size of *DA-GS-Gal4* > *Vimar/attp2* (RU−) is set as 1, and the relative ratios of other fly lines are shown. Tested fly *N* = 5. Error bars are mean ± SE. Unpaired t-test. ****P < 0.0001.

Because overexpression of *Vimar* promotes aging, we expected to find that the downregulation of Vimar prolongs lifespan and attenuates aged-related functional decline. In the *elav-GS-Gal4* > *Vimar RNAi* flies, *Vimar* RNAi expression was induced in 1-day-old or 30-day-old flies by feeding the flies RU486 (500 μM). *Vimar* RNAi expression induced in 1-day-old flies did not alter the lifespan (Fig. 3a and Supplementary Fig. 3a), although the flies with this genetic background tended to present with an inherently short lifespan (~62 days). In addition, the 55-day-old flies (in which the expression of *Vimar* RNAi was initiated when they were 1 day old and throughout adulthood, RU+ all life flies) did not present with changed climbing ability (Supplementary Fig. 3b). However, the brain ATP level, daily metabolism rates and mitochondrial membrane potential (*elav-GS-Gal4* > *Vimar RNAi* fed RU486 starting at 1 day old) all had declined compared with these parameters in the control (*elav-GS-Gal4* > *Vimar RNAi* not fed RU486) (Fig. 3b, c and Supplementary Fig. 3c, d), and the former showed elevated [Ca^2+^]_mito_ (Fig. 3c), suggesting that downregulation of Vimar from a young age may be detrimental. In contrast, *Vimar* RNAi expression (*elav-GS-Gal4* > *Vimar RNAi*) administered starting when the flies were 30 days old (RU+ half-life flies) resulted in lifespan extension, manifesting as better climbing ability than exhibited by the control flies (Fig. 3a and Supplementary Fig. 3b). In addition, the levels of brain ATP, [Ca^2+^]_mito_, the mitochondrial membrane potential, climbing ability and daily metabolism rates were all better maintained (Fig. 3b–d; and Supplementary Fig. 3b–d). MitoTracker Green staining showed mitochondrial fragmentation in the 55-day-old *elav-GS-Gal4* > *Vimar RNAi* flies that had not been fed RU486 (RU−), and feeding RU486 throughout fly adulthood (RU+ all life), did not rescue the mitochondrial morphologic defect. However, feeding RU486 late in life (RU+ half-life) rescued mitochondrial fragmentation (Fig. 3d). Collectively, these results suggest that expression of *Vimar* RNAi starting at middle age (30 days old) prolonged lifespan and attenuated age-related functional decline in *Drosophila*.

Vimar is a GEF associated with Miro, which functions in mitochondrial transport and the maintenance of mitochondrial calcium homeostasis^25,26^. To assess whether the detrimental effect of gain-of-function Vimar was mediated through Miro, we crossed flies carrying *Miro* RNAi or the genetic background control RNAi to *DA-GS-Gal4* > *Vimar* flies (*DA-GS-Gal4* > *Vimar/Miro RNAi* or control RNAi). In flies fed RU486 in the early adult stage (at 1 day old), the transcripts of Vimar and Miro were altered as expected (Supplementary Fig. 3e, f). Staining with MitoTracker Red revealed that *Miro* RNAi rescued the mitochondrial fragmentation that had been induced by *Vimar* overexpression in 40-day-old flies (Fig. 3e), and this outcome was associated with a prolonged lifespan (Supplementary Fig. 3g). These results suggest that the premature aging induced by *Vimar* overexpression depended on Miro function. In support of this conclusion, mitochondrial morphology was shown to better maintained in the 75-day-old flies (*DA-GS-Gal4* > *Vimar/Miro RNAi* fed RU486) than in the control flies (*DA-GS-Gal4* > *Vimar/Miro RNAi* not fed RU486) (Fig. 3f).

Together, these results indicate that Vimar upregulation in middle age (30 days old) may have promoted a different state of brain aging that depended on Miro in wild-type *CS* flies.

### RAP1GDS1, the mammalian homolog of Vimar, plays a conserved function in cultured cells

**Fig. 4:**
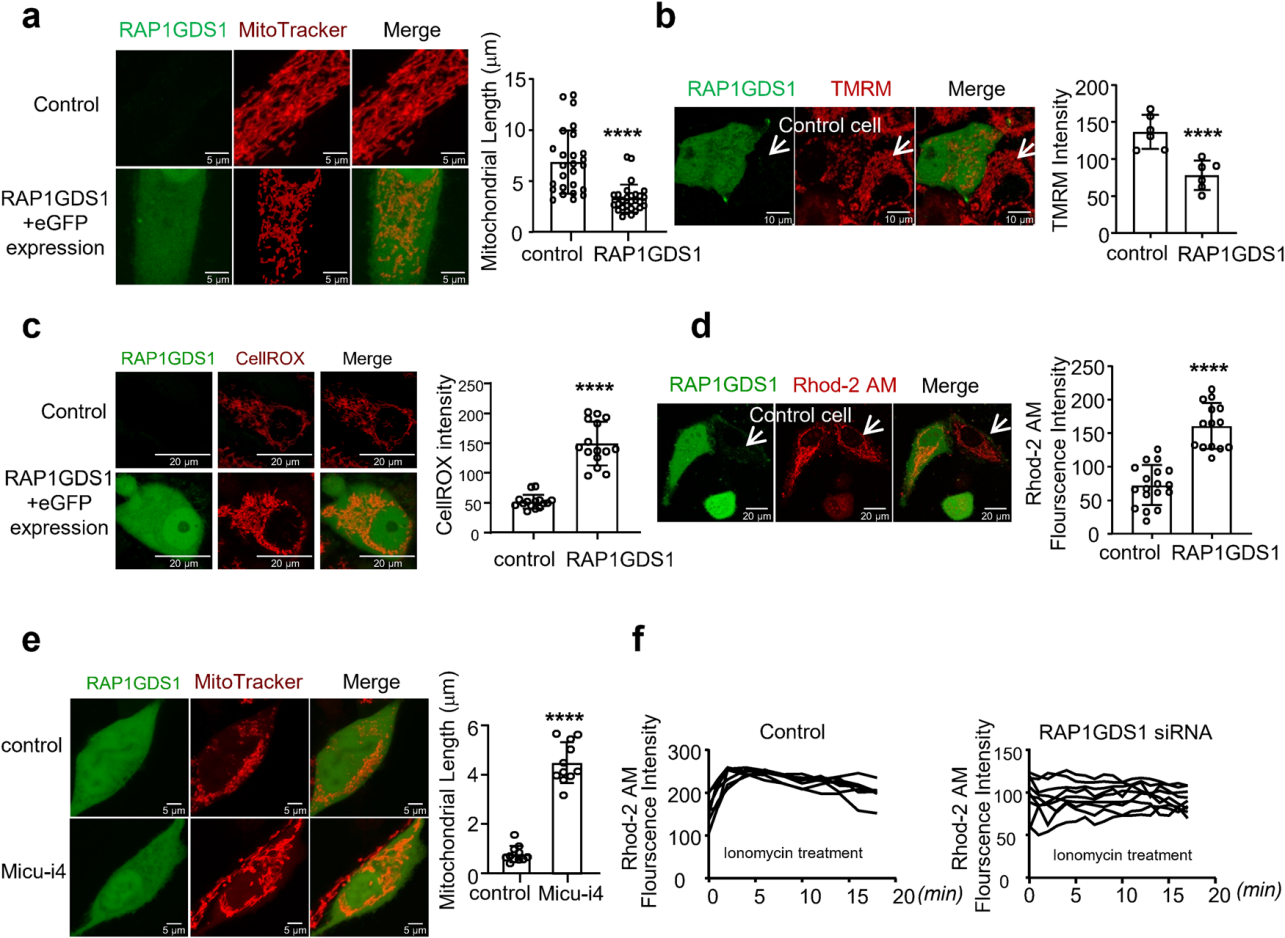
RAPGDS1 function on mitochondria in cultured cells. The U87-MG cells were transfected with a Pljm1-RAP1GDS1-eGFP vector which expresses *RAP1GDS1* and eGFP independently. **a** Representative live image of mitochondrial morphology by Mito Tracker red. The green channel is RAP1GDS1; the red channel is mitochondria. Scale bar, 5 μm. The bar graph is the mitochondrial length quantified by ImageJ. Cell number *N* = 26 (control, without *RAP1GDS1* expression) and 28 (test, with *RAP1GDS1* expression). Unpaired t-test. ****P < 0.0001. **b** Representative live image of mitochondrial membrane potential stained by TMRM. Scale bar, 10 μm. GFP positive cells are *RAP1GDS1* overexpressed. GFP negative is the control cells. The red intensity of TMRM was quantified by ImageJ. *N* = 5 (Control), 6 (*RAP1GDS1* overexpression). Unpaired t-test. ****P < 0.0001. **c** Representative live image of ROS level stained by CellROX. Scale bar, 20 μm. The red intensity of cells was quantified by ImageJ. *N* = 15. Unpaired t-test. ****P < 0.0001. **d** Representative live image of mitochondrial calcium level stained by Rhod2-AM. Scale bar, 20 μm. In the same field, the GFP positive cells represent *RAP1GDS1* overexpression; GFP negative cells are controls. Intensity of the red signal was quantified by ImageJ. *N* = 17 (control), 14 (*RAP1GDS1* overexpression). Unpaired t-test. ****P < 0.0001. **e** Representative live image of mitochondrial morphology stained by Mito tracker red. The green channel is RAP1GDS1; the red channel is mitochondria. Scale bar, 5μm. Mitochondrial length was quantified by ImageJ. *N* = 11. Error bars are mean ± SE. Unpaired t-test. ****P < 0.0001. **f** The intensity of Rhod2-AM change of U87-MG cells treated with ionomycin. Each trace represents mitochondrial calcium level changes after ionomycin treatment. For control and *RAP1GDS1* siRNA cells, *N* = 7 (control), *N* = 8 (*RAP1GDS1* siRNA). Unpaired t-test. *P* < 0.0001.

Our previous study showed that the mouse homolog of Vimar, RAP1GDS1, functions as the GEF for mammalian Miro1 and regulates mitochondrial morphology^11^. In this study, we asked whether overexpression of *RAP1GDS1* induces mitochondrial dysfunction similar to that induced by Vimar. The pLJM1-RAP1GDS1-eGFP vector expresses *RAP1GDS1* and *eGFP* driven by separate promoters. It was transfected into U87-MG cells, a human glioma cell line. Compared to the control cells, cells transfected with pLJM1-RAP1GDS1-eGFP exhibited swollen mitochondria (Fig. 4a) associated with reduced mitochondrial membrane potential, as assessed by TMRM staining, and increased mitochondrial ROS levels, as assessed by CellROX assay (Fig. 4b, c). *RAP1GDS1* overexpression also induced an increase in the [Ca^2+^]_mito_ levels (Fig. 4d). Because Miro1 regulates mitochondrial calcium uniporter (MCU) activation^25,27^, we evaluated whether *RAP1GDS1* overexpression activates the MCU channel (likely through Miro1). Indeed, both mitochondrial fragmentation and an increase in the [Ca^2+^]_mito._ (measured by Rhod-2AM assay) caused by *RAP1GDS1* overexpression was abolished by treatment with MCUi4, an inhibitor of the MCU channel^28^ (Fig. 4e). Next, we asked whether RAP1GDS1 might affect [Ca^2+^]_mito._ Level. The [Ca^2+^]_mito._ has been shown to be enhanced by ionomycin treatment^29^. Interestingly, *RAP1GDS1* downregulation by siRNA (Supplementary Fig. 4a) abolished both the [Ca^2+^]_mito_ increase (Fig. 4f) and mitochondrial fragmentation induced by ionomycin (Supplementary Fig. 4b). *RAP1GDS1* siRNA did not affect mitochondrial morphology under normal conditions (Supplementary Fig. 4c). Using the SK-N-SH human neuroblastoma cell line, we observed a similar effect of *RAP1GDS1* overexpression and RNAi on mitochondrial function (Supplementary Fig. 4d–g), suggesting that the RAP1GDS1 effect on mitochondria is not cell-type specific. Together, these results indicate that RAP1GDS1 mediates a [Ca^2+^]_mito_ increase via unknown mechanisms.

### Increased Miro1/RAP1GDS1 complex levels in mouse brain aging

Brain aging in mice is very similar to that in humans; mice can be classified by age as juveniles (<1 month (m)), young adults (1–4 m), mature adults (4–10 m), late adults (10–14 m), old (14–24 m) and senescent (>24 m)^30^. RAP1GDS1 (RAP1, GTP-GDP dissociation stimulator 1) is the mammalian homolog of *Drosophila* Vimar, which functions as a GEF for several small GTPases, including RHOA, RAC1, KRAS and Miro1; RAP1GDS1 is also known to regulate cell migration and proliferation through its associations with DNA synthesis, molecular chaperone functions and protein posttranslational modifications^11,12^.

**Fig. 5:**
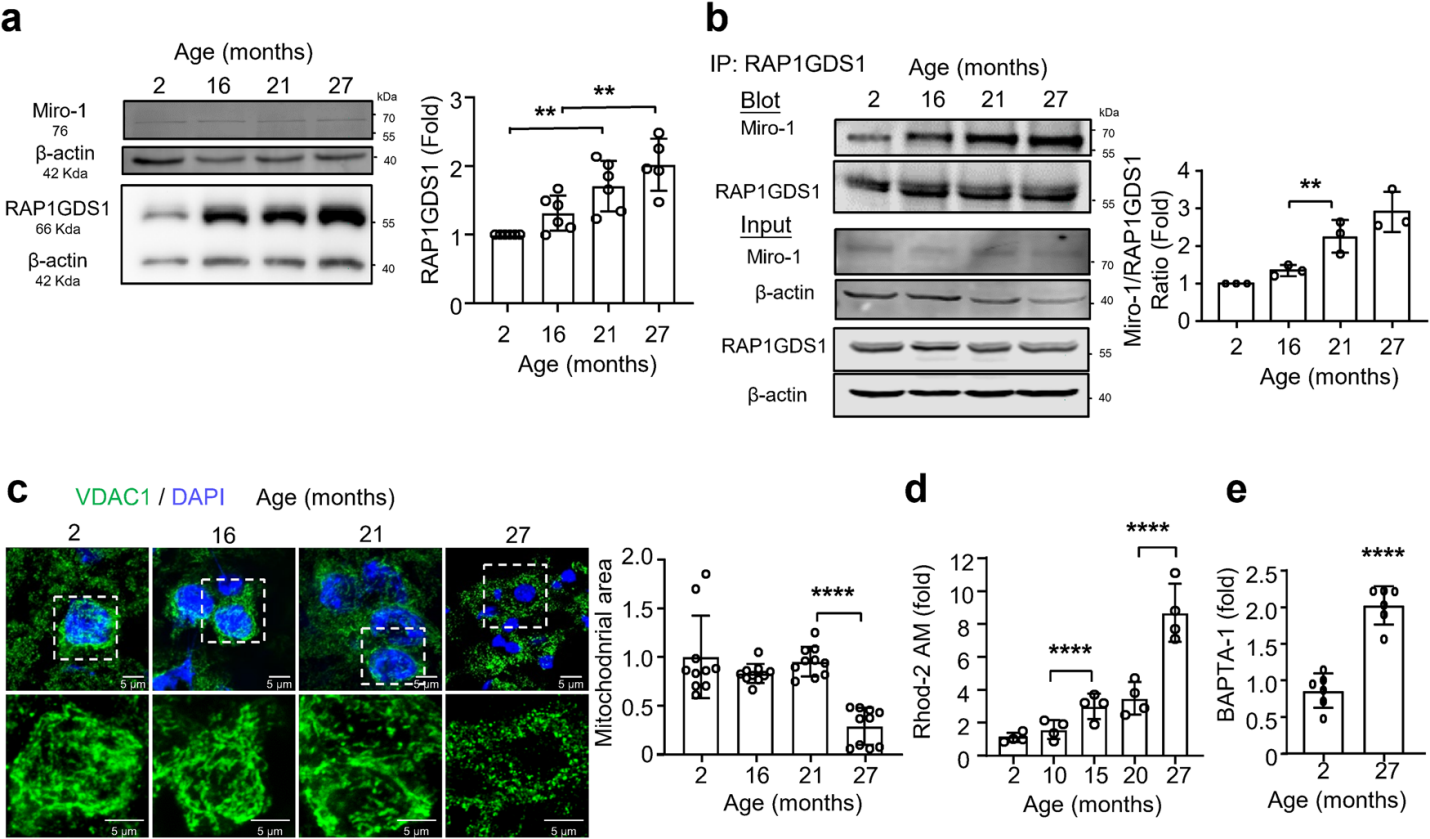
RAP1GDS1/Miro1 and mitochondrial functional change mouse brain aging. **a** Western blot analysis of RAP1GDS1 (antibody from Abcam #ab188020) and Miro1 change during aging in the brain tissue of *C57* mice. β-actin is the protein loading control. Uncropped blots are shown in Supplementary Fig. 8. With the 2 month-old sample set as 1. *N* = 6. One-way ANOVA with Tukey’s post hoc test. 2vs21, *P = 0.023; 2vs27, ****P < 0.0001; 16vs27, **P = 0.0035. **b** Co-Immunoprecipitation of RAP1GDS1 and Miro1 during aging. IP with RAP1GDS1 antibody (Santa #sc39003) to pull down Miro1. Total protein input is shown as the protein loading control. Uncropped blots are shown in Supplementary Fig. 8. The ratio of Miro1/RAP1GDS1 from the 2 month-old sample is set as 1, and the relative level of other samples are shown. *N* = 3. One-way ANOVA with Tukey’s post hoc test. **P = 0.0056. **c** Representative mitochondrial morphology stained by VDAC1 in neurons of prefrontal cortex. Scale bar, 5 μm. Mitochondrial size changes in brain neurons during aging. 2 month-old sample is set as 1. *N* = 10. One-way ANOVA with Tukey’s post hoc test. ****P < 0.0001. **d** Brain mitochondrial calcium concentration ([Ca^2+^]_mito._) change during aging. The intensity of Rhod2-AM indicates [Ca^2+^]_mito_ level. Each mouse brain quantified 20 neurons, the Rhod-2AM intensity from 2 month-old sample was set as 1, and the ratios of others are shown. *N* = 4. One-way ANOVA with Tukey’s post hoc test. ****P < 0.0001, ****P < 0.0001. **e** Cytosolic calcium ([Ca^2+^]_cyto_) change stained by BAPTA-1 AM in young (2 month-old) and senescent (27 month-old) mice. The BAPTA-1 AM intensity of 2 month-old is set as 1. *N* = 6. Error bars are mean ± SE. Unpaired t-test. ****P < 0.0001.

To evaluate whether the *RAP1GDS1* expression pattern is similar to that of Vimar in the life course of mice, we quantified *RAP1GDS1* and Miro1 transcripts in samples of brains from 2-, 10-, 16-, 21- and 27-month old mice by qRT‒PCR. The results showed that 27month-old mice showed significantly higher expression of *RAP1GDS1* and Miro1 than the younger mice (Supplementary Fig. 5a). We also quantified several genes known to regulate mitochondrial fusion and fission, including MFN1, MFN2, OPA1 and DRP1, and found no significant alterations in their expression (Supplementary Fig. 5a). The protein levels of DRP1, caspase 3 and cleaved caspase 3 were also unaltered (Supplementary Fig. 5b). Furthermore, we measured the protein levels of RAP1GDS1 and Miro1 during aging. The results showed that the expression of *RAP1GDS1*, but not that of *Miro1*, was increased in aged mice (Fig. 5a). Because Miro1 is anchored to the outer mitochondrial membrane, we extracted mitochondrial proteins and found that Miro1 was not enriched in the mitochondrial extracts obtained from the old mice (28 month-old) but was enriched in the young adult mice (2 month-old). In contrast, RAP1GDS1 protein levels in the old mice were higher than those in the young mice, in both the cytosol and mitochondria (Supplementary Fig. 5c). Furthermore, we assessed the interaction between Miro1 and RAP1GDS1 during aging. Using an anti-RAP1GDS1 antibody to perform immunoprecipitation (IP), we found that more Miro1 was pulled down in samples from older mice (Fig. 5b), indicating that Miro1/RAP1GDS1 gained functionality in mice that had passed middle age. To assess mitochondrial morphological changes during aging, we injected an AAV expressing DsRed specifically into the mitochondria of neuronal cells (AAV-BBB-mito-DsRed)^31^. The results showed that the mitochondria in the cortical neurons were smaller and fragmented in older cells (from 27 month-old mice) (Supplementary Fig. 5d). After staining with the outer mitochondrial membrane protein VDAC1, we obtained a similar result (Fig. 5c). In addition, the brain ATP level, CS activity and mtDNA content declined in the middle aged or late aging stage (27 month-old mice) (Supplementary Fig. 5e–g). Moreover, both the [Ca^2+^]_cyto._ assessed by BPAP-2 and [Ca^2+^]_mito._ accessed by Rho2-AM were greatly increased in the late adult mice (27 month-old) compared to young adult mice (2 month-old) (Fig. 5d, e). Collectively, these results indicate that accelerated brain aging in mice likely starts in middle age (21 month-old) with an increase in the RAP1GDS1/Miro1 interaction and that severe aging-related brain function decline starts at senescent age (27 month-old) and is associated with mitochondrial fragmentation.

### Premature aging is caused by *RAP1GDS1* overexpression in mice

**Fig. 6:**
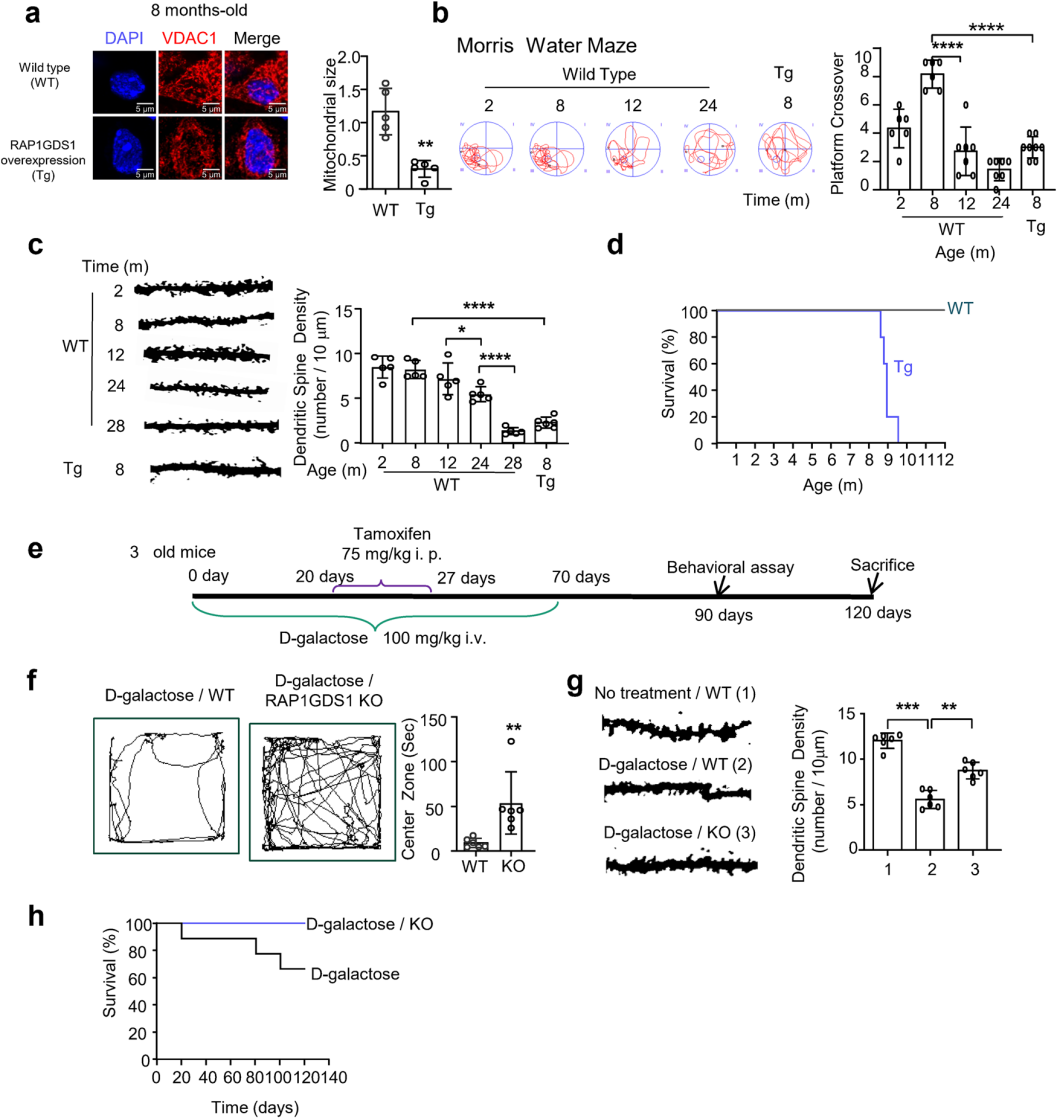
Effect of *RAP1GDS1* transgene and knockdown on mouse brain aging. **a** Immunostaining by VDAC1 to stain mitochondria in the outer molecular layer of prefrontal region. Control (8 month-old) and *RAP1GDS1* overexpression (8 month-old) is shown(left). Scale bar, 5 μm. The average mitochondrial size of control is set as 1, and the ratio of *RAP1GDS1* overexpression is shown (right). Each mouse quantified 20 neurons. *N* = 5. Unpaired t-test. **P = 0.008. **b** The water Morris maze assay. Representative traces of mice with different age and *RAP1GDS1* overexpression (8 month-old) of Morris water maze. The number of crosses of the platform in each mouse. *N* = 6, 6, 7, 7, and 8 (from left to right). One-way ANOVA with Tukey’s post hoc test. ****P < 0.0001, ****P < 0.0001. **c** Representative micrograph of Golgi staining to display the dendritic spines intensity in neurons in the outer molecular layer of prefrontal region. For wild type mice at different ages, *N* = 5. For *RAP1GDS1* overexpression mice at 8 month-old, *N* = 6. One-way ANOVA with Tukey’s post hoc test. ****P < 0.0001, *P = 0.0383, ****P < 0.0001. **d** The survival curve of wild type and *RAP1GDS1* overexpression mice. *N* = 5. long-rank(mantel-Cox) test. *P* = 0.0027. **e** Schematic time course of this experiment. 3 month-old heterozygous knockdown of *RAP1GDS1* (MAP2-Cre-ERT2^+/−^; *RAP1GDS1*^+/−^) was injection D-galactose (100 mg/kg i.v.) from day 1 to day 70. At day 20, *RAP1GDS1* knockdown was induced by tamoxifen (75 mg/kg i.p.) for 7 days. Behavioral assays were performed at day 90. These animals were sacrificed at day 120. **f** Representative traces of the open filed assay. Quantification of mouse stayed in the center zone, *N* = 6. Unpaired t-test. **P = 0.0022. **g** Representative micrograph of Golgi staining to display the dendritic spines intensity in neurons in the outer molecular layer of prefrontal region. *N* = 6. Error bars are mean ± SE. One-way ANOVA with Tukey’s post hoc test. ***P = 0.0004, **P = 0.0081. **h** Survival curve. Wild type mice treated with D-galactose, *N* = 9. Heterozygous *RAP1GDS1* knockdown mice treated with D-galactose, *N* = 8. long-rank (mantel-Cox) test. *P* = 0.0285.

To further study the effect of *RAP1GDS1* overexpression on aging, we generated conditional *RAP1GDS1***-**overexpressing mice (MAP2-Cre-ERT2^+/−^;*RAP1GDS1*^ox/+^ mice) (Supplementary Fig. 6a). Map2 was the neuron-specific promoter used to link a mutated estrogen receptor (ERT) with a Cre recombinase. Without tamoxifen induction, Cre-ERT2 is inactive in the cytoplasm. Injected tamoxifen produces 4-hydroxytamoxifen, which binds to ERT, allowing Cre-ERT2 to enter the nucleus and promote Cre recombinase expression. Cre recombinase deleted the stop codon elements flanked by LoxP in the *RAP1GDS1* transgene insertion, allowing *RAP1GDS1* expression. As expected, mitochondrial fragmentation was observed in the 8 month-old transgenic mice (Fig. 6a). Furthermore, the brain ATP level and CS activity were significantly decreased in *RAP1GDS1*-overexpressing mice compared to age-matched wild-type mice (Supplementary Fig. 6b, c). In addition, compared to that in 2-, 8-, 12-, 24- and 28 month-old wild-type C57BL/6J mice, *RAP1GDS1*-overexpressing mice showed a premature decline in behavioral performance, including nest building, speed and endurance time on a rotarod, forelimb strength and water maze memory behaviors^32–34^ (Supplementary Fig. 6d–f; and Fig. 6b). These results of aging decline in the wild type C57BL/6J mice were consistent with findings by other researchers^32^. Next, we assessed the dendritic spine density in the prefrontal cortex, which has been considered a brain aging marker^35^. The results showed that the dendritic spine density in the 8 month-old *RAP1GDS1*-transgenic mice was even lower than that in the 24 month-old wild-type mice (Fig. 6c). More important, the lifespan of the *RAP1GDS1*-transgenic mice was much shorter than that of the wild-type mice (Fig. 6d). Collectively, these results suggest that overexpression of *RAP1GDS1* in neurons induces premature aging in mice.

### *RAP1GDS1* loss of function delays aging-related functions in mice

Our data indicated that RAP1GDS1/Miro1 negatively impacted mitochondrial morphology and respiration after mice and flies reached middle age. We expect that downregulation of *RAP1GDS1* in the late aging stage may be protective. To quickly assess this hypothesis with mice, brain aging was induced by D-galactose injection, which caused dysfunctional mitochondrial respiration and increased ROS level. The Morris water maze is a commonly test used to determine brain aging^36^. For these experiments, mouse aging was induced by D-galactose injection (100 mg/kg)^37,38^. The time course of the experiment is outlined (Fig. 6e). Briefly, 3 month-old mice were injected daily with D-galactose for 70 days. After D-galactose application for 20 days, heterozygous *RAP1GDS1* knockdown (MAP2-Cre-ERT2^+/−^;*RAP1GDS1*^ko/+^) was induced by the injection of tamoxifen for 7 days. We verified the knockdown effect (Supplementary Fig. 6g). At 90 days (without D-galactose application for the previous 20 days), we examined aging-related behavioral performance of the mice with open-field, nest building, rotarod, and novel object recognition tests. The results showed that D-galactose treatment caused behavioral decline, and these deficits were alleviated by heterozygous *RAP1GDS1* knockdown (Fig. 6f; and Supplementary Fig. 6h–j). In addition, the D-galactose-induced reduction in dendritic spine density and survival were rescued in the mice with a *RAP1GDS1* heterozygous knockdown background (Fig. 6g, h). Furthermore, the swollen mitochondrial morphology induced by D-galactose was rescued by the heterozygous knockdown of *RAP1GDS1* (Supplementary Fig. S6k), along with better maintenance of brain ATP levels and increased CS activity in the frontal cortex (Supplementary Fig. S6l, m). In addition, the early lethality induced by D-galactose was rescued in mice with a heterozygous *RAP1GDS1* knockdown background. These results suggested that downregulation of *RAP1GDS1* might promote a prolonged health span in mice. Although the sample was too small to draw a conclusion with confidence, this result suggests that D-galactose-induced mitochondrial dysfunction may be related to RAP1GDS1/Miro1 function during mitochondrial calcium overload. This possibility requires further investigation.

## Discussion

Under normal physiological conditions, human brain aging accelerates in the late aging stage is unclear. By using brain mapping techniques, several studies have demonstrated that global gray matter volume shows a nonlinear decline with age, showing accelerated loss in some brain regions^39,40^. For instance, 32% of gray matter density in the superior frontal sulcus is lost in people between the ages of 7 and 60 years, but only a 5% decline is observed in people between the ages of 40 and 87 years; in contrast, the gray matter density loss in the superior temporal sulcus was 12% in people between the ages of 7 and 60 years, and the loss increased, showing a 24% decline, in people between the ages of 40 and 87 years^39^. This cortical volume loss during normal aging is likely a result of neuronal shrinkage, not cell death^41^. This possibility is supported by many studies with mice, rats, monkeys and humans. In general, dendritic length, arborization, spine and synaptic density showed variable degrees of decline in most brain regions, especially the prefrontal and superior temporal cortex, and these changes were associated with reduced levels of neurotransmitters and ion channels^42^. Recent studies showed that the synaptic density in old C57BL/6J mice (14 month-old) was greatly reduced compared with that in young mice (8 month-old)^35,43^, and 18 month-old mice showed a further reduction compared with that in 14 month-old mice^35^. These studies indicate that brain atrophy and reduced dendritic span density may alter synaptic currents and impair brain function^42^. Consistent with the results of these studies, our data showed that dendritic span density in the prefrontal cortex of 24month-old mice was significantly reduced compared to that of 12 month-old mice, and the span density in *RAP1GDS1* transgenic mice was reduced prematurely. In addition, *RAP1GDS1* transgenic mice showed premature behavioral defects in nest building, rotarod and Morris water maze tests. As a home-care behavior, deficits in nest building correlate with brain damage, particularly early brain dysfunction. Nest building is a sensitive test used to evaluate age-related behavior decline^44^. The locomotor function of C57BL/6 mice shows an age-dependent decline; thus, the rotarod assay is a popular test for studying aging in mice^45,46^. The Morris water maze is one of the most commonly used behavioral tests to study normal aging, due to spatial learning behavior is mostly regulated by the dorsal hippocampus and cerebellum^46^. Our data suggest that *RAP1GDS1*-transgenic mice can be used as a model of senescence^47^.

Based on daily metabolic rate data in people^9^, accelerated aging may start in people in their 60 s, which is approximately the final 1/3 of the lifespan. In our study, the elevated expression of Vimar was observed in 50 day-old flies and was associated with a significant decline in the brain ATP level, followed by cytosolic calcium overload in 60 day-old flies and mitochondrial calcium overload and fragmentation in flies ~70 day-old. Considering the 82 days *CS* fly lifespan, the final 1/3 of the lifespan is ~55 days, which is the starting point of accelerated brain aging. As the median lifespans of female and male C57BL/6J mice are ~866 days (~29 months) and 901 days (~30 months)^48^, we observed a significant increase in the interactions between Miro1 and RAP1GDS1 in C57BL/6J male mice at the age of 21 months. Similar to *Drosophila*, mitochondrial fragmentation occurred later in life, at the age of 27 months. A study comparing young (average age of 38 years old) and old (average age of 78 years old) biopsy samples from human muscle showed that *RAP1GDS1* expression was increased by 3.16-fold in the old samples^49^. The time point when *RAP1GDS1* expression initially increasing in mice is unclear and is worthy of future investigation. Overall, these results suggest that gain-of-function of Vimar/RAP1GDS1 after middle age (2/3 of the lifespan) may initiate a different rate of aging decline in wild-type flies and mice.

In adult flies, the conditional overexpression of Vimar promoted premature aging, including a shortened lifespan, reduction in locomotive ability, decreased brain ATP level and reduced mitochondrial metabolism. This suggests that gain-of-function *Vimar* during aging after middle age is detrimental. Furthermore, knocking down *Vimar* in flies that have passed middle age extended the fly lifespan, which was manifested as attenuated dysregulated mitochondrial morphology and function. Interestingly, downregulation of *Vimar* from a young age did not increase lifespan, indicating that Vimar is required for normal physiological function in flies at a young age. In support of this finding, a loss-of-function *RAP1GDS1* mutant was identified in patients that was associated with developmental delay, hypotonia and cognitive deficits, indicating that RAP1GDS1 may play an important role in development^12^. Transcriptional changes associated with brain aging have been documented in diverse organisms. However, the mechanistic insight into the regulation of gene expression during aging is unclear. An interesting study based on a single-cell transcriptome analysis of the *Drosophila* brain demonstrated that aged neurons showed reduced expression of genes involved in oxidative phosphorylation and cell shrinkage compared to that in young neurons (45 day-old vs. 3 day-old flies)^50^. The mechanism underlying increased *Vimar* expression in brain aging is still unclear.

Our previous study showed that Vimar physically interacted with Miro and that *Vimar* loss-of-function suppressed mitochondrial enlargement caused by Miro gain-of-function mutation, suggesting that Miro function depends on Vimar and that RAP1GDS1 exhibits a similar role to Miro1 in mammalian cells^11^. In *Drosophila*, Miro gain-of-function mutation promotes calcium transport from the endoplasmic reticulum (ER) to mitochondria by increasing the contact sites between the ER and mitochondria and requires inositol 1,4,5-trisphosphate receptor (IP3R) on the ER membrane, voltage-dependent anion-selective channel (VDAC) in the mitochondrial outer membrane (MOM), and mitochondrial Ca^2+^ uniporter (MCU) in the mitochondrial inner membrane^19^. A study in mammalian neurons demonstrated that the N-terminal MCU colocalized with Miro1 at the mitochondrial outer membrane, and cytosolic calcium overload induced the cleavage of the N-terminal MCU and resulted in the disassociation of MCU and Miro1, which caused an increase in mitochondrial calcium mediated via an unknown mechanism. Our data suggest that overexpression of *Vimar* or *RAP1GDS1* promoted mitochondrial calcium increase. Because Miro1 induces mitochondrial calcium elevation mediated via MCU through an unknown action^25,26^, RAP1GDS1 might affect mitochondrial calcium through its interaction with Miro1. Nevertheless, our data suggest that *Vimar* gain-of-function acquired after flies reach middle age causes mitochondrial calcium overload, which accelerates brain aging.

This research has limitations. We have not obtained lifespan data after obtaining *RAP1GDS1*-conditional knockdown data from analyses on middle aged samples. We do not know the mechanisms of *Vimar/RAP1GDS1* overexpression or their modes of action to induce mitochondrial calcium overload. Moreover, we do not know whether DRP1 contributes to the mitochondrial morphological changes induced by *Vimar* or *RAP1GDS1* overexpression. In the lifespan studies performed after *RAP1GDS1* was overexpressed or knocked down in mice, the sample size was small; therefore, interpretation of the results is only suggestive. In addition, how RAP1GDS1 exerts its GEF activity on Miro1 and whether this GEF function is altered during aging remain unknown.

This study demonstrated that increased *Vimar* expression in the brain of flies that have reached middle age serves as a different stress that promotes brain aging. This alteration is conserved in the mouse brain, which shows increased Miro1/RAP1GDS1 interactions starting during middle age. This stress promotes mitochondrial calcium overload and ultimately mitochondrial fragmentation in approximately the final 1/10 period in the lifespan, which begins on Day 75 in the 82-day lifespan of *Drosophila* and in Month 27 in the 30 month lifespan of mice. If the human lifespan is ~90 years, middle age-related brain aging may start at 60 years (1/3 of the last lifespan), and accelerated brain aging occurs at 81 years old (1/10 of the last lifespan).

## Methods

### Animal care and transgenic mice

All animal procedures were approved by the institutional animal care committee (AEEI-2019-048). Animals were housed with a 12:12 h light: dark cycle with food and water available ad libitum. *RAP1GDS1*-conditional overexpression and knockdown mice were generated by Shanghai model organisms. C57BL/6-*Map2*^*em1(2A-CreERT2)Smoc*^ mice were purchased from Shanghai model organisms. *RAP1GDS1*-conditional knock-in/down transgenic mice were crossed with C57BL/6-*Map2*^*em1(2A-CreERT2)Smoc*^ mice. Tamoxifen (75 mg/kg i.p. for 7 days) injection thus induces activation of neuronal CreERT2 resulting in a gain of function or null *RAP1GDS1* expression. All mice were genotyped via PCR analysis of tissue biopsy samples and exam the protein expression by western blot of brain tissue.

### Fly stocks

The following fly stocks were obtained from Bloomington Drosophila Stock Center: *w*^*1118*^ (#3605), *CS* (#64349), *UAS-mito-GFP* (#8442), *Elav-GS-Gal4* (#43642), *Daughterless (DA)-GS-Gal4*, *Appl-Gal4* (#34040). Tsinghua Fly Center: *Vimar RNAi* (TH1142), *Miro RNAi* (TH2781), TRip background control (*attp2*, TB00072). We generated the following lines: *hs-GluR1*^*Lc*^, *UAS-Vimar/tb*, and *UAS-Vimar/cyo*. For all experiments, flies were kept at 25 °C with a 12:12 h light/ dark cycle and constant humidity (65%) on the standard sugar−yeast−agar medium (15 g/l agar, 50 g/l sugar, 100 g/l autolyzed yeast, 6 g/l nipagin and 3 ml/l propionic acid). Flies were raised at standard density in 100 ml bottles unless otherwise stated. All experiments of survival assay and behavior test were carried out with male and female files (10:10 in each tube). Female flies were used in all confocal microscopy imaging experiments.

### Daily energy expenditure

This experiment was followed a previous protocol^13^. Briefly, 3–5 flies were put into a small chamber with a micropipette contain soda lime that could absorbed CO_2_, allowing the pipette to submerge into the colored solution. Then take a photo of the chamber to make sure that the level of liquid within each micropipette and the scale are visible, after 20–30 min, take another picture, measured the distance (Δd) that the liquid traveled from a determined reference spot in images taken at the beginning (d1) and end of experiment (d2), and then calculate the amount of produced CO_2_ (μl/hour/fly).

### ATP measurement

ATP concentration was determined using the ATP Assay Kit (ab83355, abcam). Briefly, twenty fly heads were homogenized in 100 μl ice-cold ATP assay buffer with a Dounce Homogenizer. Lysate was then centrifuged for 5 min at 13,000 × *g* at 4 °C. Add 50 µl Reaction Mix to 50 μl sample wells. Incubated at room temperature for 30 min and then measured on a microplate at OD 570 nm. Total protein amount was measured using the pierce™ BCA protein assay kit (#23225, Thermo Fisher). The ATP level in each sample was normalized to the total protein amount.

### Citrate synthase activity assay

40 fly heads or 100 mg mice brain tissue was homogenized with 200 μl extraction buffer containing 20 mM HEPES (PH7.2), 1 mM EDTA and 0.1% triton X-100, and centrifuge for 15 min at 4000 rpm at 4 °C. Add 20 μl supernatant to 70 μl working buffer (100 mM Tris-Hcl, PH 8.0 ;100 μM DTNB; 50 μM acetyl coenzyme A; 0.1% Triton X-100; 250 μM Oxaloacetate), mix immediately and read by OD absorbance at 412 nm for 5 min per 10 s; and then normalized to total protein amount^1^.

### Fly behavioral assays

We performed climbing agility assay to measure fly locomotor abilities. Briefly, adult flies were gently tapped to the bottom of a modified 25 ml climbing tube and their climbing progress was recorded after 45 s. Three to six populations of flies (about 60–180 flies used for every test) were assessed, and for each population, flies were examined three times per experiment. The recorded values were used to calculate the average performance index.

### Live ROS detection

Fly brains were dissected in ice-cold Schneider’s medium (according to the manufacturer’s introduction from sigma) and stained with 100 nM CellROX™ (C10443, Thermo) in Schneider’s medium for 30 min at room temperature in darkness. Then permeabilize brains with 30 μg/ml digitonin (#300410, sigma) for 2 min and wash 3 times with Schneider’s medium. Image immediately in a chamber on a slide with a coverslip. Cells were washed by PBS, incubated with 50 nM CellROX™ (Thermo Fisher) for 30 min, and washed 3 times with PBS and finally imaged immediately. Leica SP8 laser scanning confocal microscope equipped with a 63 NA oil objective as Z stacks was used, with identical imaging parameters among different phenotypes in a blinded fashion. The total intensity of each individual sample was measured by Image J (Fiji) and normalized to the background intensity.

### Cell culture and transfection

SK-N-SH, U87-MG and HEK-293T were purchased from ATCC (American Type Culture Collection) with order number HTB-11, HTB-14 and CRL-111268, respectively. The U87-MG and SH-SKN cells were cultured in the DMEM medium (Gibco) supplemented with 10% FBS (Gibco), streptomycin (0.1 mg/mL) and penicillin (0.06 mg/mL) (Gibco). Lipo8000 (Byotime) was used for transfection following the manufacturer’s protocol.

### Real-time PCR

Total RNA was extracted from about 40 heads of fly or 20 mg of mice brain tissue using TRIzol (Thermos) according to the manufacturer’s protocol. concentrations of total RNA were measured by NanoDrop 2000 (Thermos). Total RNA was then subjected to gDNA digestion using DNaseI (Byotime), immediately reversed to cDNA followed by EasyScript^®^ First-Strand cDNA Synthesis SuperMix (Transgene AE301). Real-Time PCR was performed using FX7 (Thermo Fisher Scientific) and PowerUp™ SYBR™ Green Supermix (A25742, Thermo Fisher) following the manufacturer’s protocol. The results were analyzed by the thermos express software, and relative expression level was presented as the ratio of the target gene to the endogenous gene, actin. The primer sets for the real-time PCR are listed in Supplementary Table 1.

### mtDNA contents

Total genomic DNA was isolated using Puregene Cell and Tissue Kit (QIAGEN) and was amplified using specific primers of Ms-Dloop1, Ms-COX1, Ms-ND4, Ms-16S, Ms-RNR-S, D–COX1, and D–CytB by real-time PCR using the Power SYBR Green RT-PCR kit (Applied Biosystems). The mtDNA copy number was calculated using Ms-RNR-S and D-RPL32 amplification as a reference respectively for mouse and Drosophila nuclear DNA content. The primer sets for the real-time PCR are listed in Supplementary Table 2.

### Western blot

For *Drosophila*, 40–60 heads were homogenized in ice-cold Cell lysis buffer for Western and IP (P0013, Byotime) containing 1×PMSF and Complete™ Protease Inhibitor Cocktail (#46931, Roche) for 30 min on ice, then centrifuge at 12,000 × *g* 4 °C for 15 min. Add 4× loading buffer into the supernatant. Boiled for 5 min before loaded into BeyoGel™ Plus Precast PAGE Gel (P0523FT, Byotime). BeyoGel™ Plus SDS-PAGE Hepes Electrophoresis Buffer (P0052, Byotime) was used for electrophoresis. After electrophoresis, PVDF membrane (0.2 μm, Milipore) was used for wet transfer with Western Transfer Buffer (P0021, Byotime). Transferred membranes were blocked in TBST(TBS with 0.05% Tween-20 and 5% milk) for 1 h at room temperature, and then diluted following primary antibodies with QuickBlock™ Primary Antibody Dilution Buffer (P0256, Byotime) for Western Blot at 4 °C overnight; Vimar (generated ourselves, Rabbit, 1:2000), β-actin (HC-201-01, Transgen mouse, 1:2000; 66009-1 g, Proteintech, Rabbit, 1:5000; ab8224, abcam, Mouse, 1:2000). The Transgen antibody of β-actin detected a non-specific band around 30 kDa in fly samples. Rhot1 (sc35928, santa, Mouse, 1:500), RAP1GDS1 (10377-AP, CST, Rabbit, 1:1000; ab224413, abcam, Rabbit, 1:500; sc39003, santa, Mouse, 1:250). Drp1 (ab184247, abcam,Rabbit, 1:1000). Rhot1 (Abcam, ab188029 1:2000; Santa Cruz, SC398520, 1:1000), VDAC1 (Abcam, ab186321 1: 2000 (WB),1: 250 (IF)). Secondary antibodies were HRP-conjugated goat anti rabbit/mouse (ab205718, ab205719, abcam, 1:10000) for 1 h at room temperature. Immunoblot Western HRP Substrate ECL reagents (WBKLS0500, Merk) were used for chemiluminutesescence. Membranes were exposed by Vilber Chemiluminutesescence imaging system Fusion FX7 and quantification was carried out with its own system software. Experiments were repeated more than three times.

### In vivo binding assay

20 mg of brain tissue from mouse prefrontal cortex were homogenized in Tissue lysis buffer for Western and IP (P0013, Byotime) containing 1×PMSF and cOmplete™ Protease Inhibitor Cocktail (#46931, Roche). Incubate on ice for 30 min, and centrifuge at 4 °C 12,000 × *g* for 20 min, meanwhile measure the total protein concentration by pierce™ BCA protein assay kit (#23225, Thermo Fisher). Dilute the lysis to 2 mg/ml with lysis buffer and part of the total protein lysate was reserved as “Input”. Incubate 500 μl lysis with 10 ul RAP1GDS1 antibody (Sc39003 Santa Cruz) or equal-quality normal mice IgG (vk312220, Thermo Fisher) for about 10 h on a nutator at 4 °C. Transfer the mix to 50 μl pre-washed protein A/G agarose for 8 h on a nutator at 4 °C, then centrifuge the mix at 4000 × *g* for 5 min at 4 °C and finally wash the beads five times with lysis buffer. Residual buffer was removed from the last wash and the beads-antibody complex was mixed with 100 μl 1×loading buffer and loaded into SDS-PAGE. For each running, ~10% of total lysates (Input) and 20% of total immunoprecipitated proteins were loaded.

### TUNEL assay

Detection of apoptotic cells was performed using TUNEL Apoptosis Detection Kit (Vazyme, A111-02, China) according to the manufacturer’s instructions. Fly brain tissue was cyrosectioned, and the slides were air-dried for 20 min; then fixed with 4% PFA for 30 min at room temperature. Then, slides were washed twice with PBS for 5 min. Incubate slides in a 20 µg/ml Proteinase K in PBS for 10 min. Repeat the wash process. For positive control, 20 U/ml DNase I were required to treat them for 10 min at room temperature. After equilibration buffer treatment for 10–30 min, a total volume (50 µl) of detection buffer (34 µl ddH2O + 10 µl 5× Equilibration Buffer + 5 µl FITC-12-dUTP Labeling Mix + 1 µl Recombinant TdT Enzyme) were added to each slide protected from light for 1 h. Finally, 2 μg/ml DAPI were used to visualize the nucleus. Images were obtained with an Axio Examiner (ZEISS).

### Live mitochondrial morphology imaging

Transgenic *(Appl* > *mitoGFP*; *Mhc* > *mitoGFP*) fly brains were dissected in ice-cold Schneider’s medium (according to the manufacturer’s introduction from sigma), and for other transgenic fly lines, brains were dissected out and incubated with 200 nM Mito Tracker (m7513/m7514, Thermo Fisher). Cells were washed with PBS to remove cell debris, and incubated with 200 nM Mito Tracker for 30 min, finally washed 3 times with PBS. Images were acquired using Leica SP8 laser scanning confocal microscope equipped with a 63 NA oil objective as Z stacks, with identical imaging parameters among different phenotypes in a blinded fashion. The length and area of mitochondrion were measured by Image J (Fiji) according to ref. ^2^, for mitochondrial size analysis of brain and muscle, every brain contain 5 independent vision window (200 × 200), and then calculated the mean size; for mitochondrial size analysis of siRNA, details described in the related figure legends.

### Live calcium concentration measure

Fly brains were dissected in ice-cold Schneider’s medium (according to the manufacturer’s introduction from sigma). For [Ca^2+^]_cyto._ measurement. The brain was stained with 7 μM Fura-2AM for 30 min at room temperature under darkness, then washed for 5 min under darkness. Image immediately by Olympus microscope (IX83) equipped with objective (UAPON40XO340-2) and DG-4 (shutter); For [Ca^2+^]_mito._ measurement, the brain stained with 7 μM Rhod-2AM. The mice brain slices were incubated in the Rhod-2AM (7 μM) or BAPTA-1 (1 μM, cytosol calcium indicator) for 1 h and then washed with artificial cerebrospinal fluid (aCSF 19 mM NaCl, 26.2 mM NaHCO_3_, 2.5 mM KCl, 1 mM NaH_2_PO_4_, 1.3 mM MgCl_2_, 10 mM glucose, 2.5 mM CaCl_2_) for 3 times. Before use, gas the aCSF solution with 5% CO_2_/95% O_2_ for 10–15 min. Then imaging with Axio Examiner (Zeiss). Cells were cultured in confocal dish, and washed with PBS. Then add 7 μM Rhod-2AM (HY-D0989, MCE) in DMEM no-phenol red in culture incubator for 1 h. Then permeabilize with 30 μg/ml digitonin (#300410, sigma) for 2 min and wash 3 times with PBS. Images were acquired using Leica SP8 laser scanning confocal microscope equipped with a 63 NA oil objective as Z stacks. After ionomycin treatment, take photo every 30 s for 40 times to monitor measure [Ca^2+^]_mito._ change, with identical imaging parameters among different phenotypes in a blinded fashion. The total intensity of each individual samples was measured by Image J (Fiji) and normalized to the background intensity.

### Live membrane potential imaging

Fly brains were dissected in ice-cold Schneider’s medium (according to the manufacturer’s introduction from sigma) and stained with 100 nM TMRM (I34361, Thermo Fisher) in Schneider’s medium for 30 min at room temperature protected from light and then washed with 30 μg/ml digitonin (300410, sigma) for 2 min and washed three times with Schneider’s medium. Cells were treated with 100 nM TMRM (I34361, Thermo Fisher) in DMEM medium with 10% fetal bovine serum (FBS, Gibco) and 1% Pen Strep (Thermo Fisher) for 30 min at 37 °C protected from light, then washed cells for 3 times and incubated with DMEM (no phenol red, 21063029, Gibco). Images were acquired using Leica SP8 laser scanning confocal microscope equipped with a 63 NA oil objective as Z stacks, with identical imaging parameters among different phenotypes in a blinded fashion. The total intensity of each individual sample was measured by Image J (Fiji) and normalized to the background intensity.

### Mitochondrial morphology imaging of mouse brain and confocal microscopy

Two methods be used for observing mitochondrial morphology in mice brain. A) injection AAV-BBB-Mito DsRed by the tail vein 100 μl/mice. After 4 weeks, mice were anesthetized with pentobarbital sodium (80 mg/ml) and perfused with ice-cold 0.9% saline, finally embedded in OCT (SAKURA#4583). Samples were frozen by liquid nitrogen & isopentane for 45 s and then cut into 100 μm slices. B) Immunostaining. Mice was anesthetized with pentobarbital sodium (80 mg/ml) and perfused with ice-cold 0.9% saline, finally embedded in OCT (SAKURA#4583). Samples were frozen by liquid nitrogen & isopentane for 45 s and then cut into 8 μm slices. The slices fixed and permeabilized by acetone for 5 min, followed by blocking with 5% donkey serum in PBS with 0.2% BSA for 1 h at room temperature. Incubate with primary antibody (VDAC1, ab14734, abcam, 1:250) in blocking solution overnight at 4 °C, then wash with PBST (0.2% Triton X-100) 3 times for 5 min. Secondary antibody (1:1000) was used for 4 h at room temperature under darkness. Images were acquired using Leica SP8 laser scanning confocal microscope equipped with a 63 NA oil objective as Z stacks, with identical imaging parameters among different phenotypes in a blinded fashion. The length and area of mitochondrion were measured by Image J (Fiji)^51^.

### Golgi staining

The experiment followed the protocol provided by FD rapid Golgi staining kit (PK401, FD). In brief, the fresh brain without any perfusion was immersed in the Golgi solution for more than 2 weeks. The brains were sectioned at 150 μm thickness in a vibrating microtome and colored following the instructions from the manufacturer and imaged under microscope after drying. Bright field images were obtained by a Zeiss Axiovert 200 M microscope with the ProRes software for the spine pictures and with the Openlab software for the dendrites. Dendrites were drawn and analyzed by using the ImageJ (FIJI) software.

### Lifespan

Flies were raised at a standard density in 200 ml bottles. After eclosion, flies were allowed to mate for 24–48 h in the bottles. Mated females were then split into vials containing SYA medium with or without compounds, with 20 flies in each vial. Adult-onset, tissue-specific RNAi was achieved as previously described. Briefly, 24–28 h after eclosion, transgenic flies were fed on SYA medium supplemented with ethanol or with mifepristone (RU486, 500 μM) to induce UAS expression from adulthood. Alternatively, flies were initially fed on normal medium for 30 days and then switched to RU486 (500 μM) supplemented food. For all non-induced control food, the same volume of ethanol was added. Flies were tipped onto fresh food and fly deaths were scored 3 times a week. The final number was the total number of dead flies. Lifespan experiments were performed at 25 °C unless otherwise stated. Data are presented as cumulative survival curves, and survival rates are compared using log-rank test.

### Nest building assay

We followed a previously reported methods. In brief, cut soft paper into 1 cm wide and weigh out about 3 g as nesting material mix paper with corn-cob liters. Each mouse was housed in a single cage. After 12 h score the nest according to a protocol^3^. To quantify the behavior, we first determined whether the paper had been touched by the mouse. If the material was untouched, the nest building score was recorded as 0. If the paper was moved, but not form a cage, receives a score of 1; If a centralized nest site is present, the nest is flat with no shallow walls receives a score of 2. A slightly cupped shape where the wall of the nest is less than 1⁄2 the height of a dome that would cover a mouse receives a score of 3. A wall that is 1⁄2 the height of a dome receives a score of 4. If the paper wall was taller than 1⁄2 the height of a dome, a score of 5 was given.

### Rotarod test

Motor behaviors of mice were assessed on a 3 cm diameter rod, elevated 35 cm above the floor using the AccuRotor Rotarod Apparatus (Pan lab, Span). Mice were trained for 3 times, and the habituation trial lasted for 5 min at 4 rpm each time. One hour after training, the speed of the rotation gradually increased from 4 to 40 rpm in 5 min. Record the maximum speed and time while mice drop.

### Grip strength test

The process was followed the protocol from Jackson lab. In short, gently pull the mouse back by its tail ensuring the torso remains parallel with the grid. Before any measurements were taken, allow both its forepaws and hind paws to attach to the grid. Record the maximal grip strength value of the mouse.

### Open field test

As previously reported^4^, motor behavior of mice was assessed in a 40 × 40 × 30 cm activity monitor (R.W.D, China) with an outlined center area (13 cm×13 cm). Each mouse was placed at the corner of box, which served as a starting point, then allowed to move freely for 5 min in the box. Time traveled in the maze and the maximum speed were measured.

### Novel object recognition

This test was assessed in an open field (40 × 40 × 30 cm) with small, plastic toys that have distinct shapes (cones, cuboid and cylinder). Mice were placed in an open field that contained 2 identical objects during training for 5 min. Two hours later, mice were placed with the familiar and a novel object for five minutes^5^. Increased percentage of time spent with the novel object is an index of improved performance in this task.

### Morris water maze test

Briefly, the mice were allowed to habituate the water maze (110 cm in diameter) at the 1st day^6^. The maze filled with opacified water was refreshed every day, and the temperature of the environment and water maintained at 19 °C to 22 °C. Mice were trained for 6 continuous day, during the training period, they were allowed to freely swim for 60 s to find the platform (10 cm in diameter), which was fixed 1 cm beneath the water surface. Mice that failed to find the platform were guided to it and allowed to stay for 30 s. After training, the platform was removed and the mice were tested in one minute. The swimming activity of each mouse was monitored using a video camera mounted overhead and was automatically recorded via Smart digital tracking system (Smart version 3.0, Panlab).

### Statistics and reproducibility

Throughout the paper, the distribution of data points is expressed as dot-plot with mean ± S.D. or survival curve, except otherwise stated. For all bar graphs throughout all figures, statistical significance was performed with unpaired *t*-test (two-tailed) between two groups. Data are presented as mean ± SE. For comparison of more than two groups, significance was determined by one-way ANOVA with Tukey’s post hoc test. Long-rank (mantel–Cox) test was used for survival analysis. Statistical analyses were performed using the Prism software (v.8.01, Graph Pad). No statistical methods were used to predetermine sample size. Experimental replicates were chosen based on the nature of the experiments. We did not exclude any data. *P < 0.05, **P < 0.01, ***P < 0.001 and ****P < 0.0001. For the number of flies used in each experiment, 15–20 adult flies with mixed sex were collected and put into a vial. 3–7 vials were tested for each condition. We tried to minimize usage of mice for the animal welfare. Typically, we tested 6–8 male mice for each condition, especially for the experiments have reached statistical significance with low animal number.

## Supplementary information

Supplementary Information

Description of Additional Supplementary Files

Supplementary Data 1

## Data Availability

All materials and data are available upon request. Source data underlying figures are provided in Supplementary Data 1. Uncropped blots are presented in Supplementary Figs. 7–14.
