# Supplementary material for: Vimar/RAP1GDS1 promotes acceleration of brain aging after flies and mice reach middle age
Source: Commun Biol. 2023 Apr 15;6:420. doi: 10.1038/s42003-023-04822-1 (PMC10105717; doi:10.1038/s42003-023-04822-1)
Supplement: Supplementary file 1 — Supplementary Information [file 42003_2023_4822_MOESM1_ESM.pdf]

Supplementary information

Supplementary Table 1 List of primers for Real-Time PCR

Vimar forward	5’ GCACCCGCCGAACAGA 3’
Vimar reverse	5’ TGCGATCGTAGTCTTGCGTTA 3’
D-Miro forward	5’ GAGCGGCATATCTCACTTGGA 3’
D-Miro reverse	5’ GCTGCGACGCCGTGTAC 3’
D-Mitofusion 1 forward	5’ CCGGAATTCCCAGCACAA 3’
D-Mitofusion 1 reverse	5’ TTATCCAAATGGGCACATAACG 3’
D-OPA1 forward	5’ CCTTCAGCGTGATAACGTGGAT 3’
D-OPA1 reverse	5’ CCGGGAACCACGTTTGC 3’
D-Mito fusion 2 forward	5’ CATTTACGAGCGGATCACCATA 3’
D-Mito fusion 2 reverse	5’ GCGTTCGGTGAGCAGGTT 3’
D-DRP1 forward	5’ GCTGCAGATCATCACGAAGTTC 3’
D-DRP1 reverse	5’ CCGTGCCCTCGATTGTG 3’
D-Actin forward	5’ GCTCCTCGTTGGAGAAGTCCTA 3’
D-Actin reverse	5’ CGTTGCCGATGGTGATCA 3’
Ms-RAP1GDS1 forward	5’ TGTCGCCCTGGCACTGATA 3’
Ms-RAP1GDS1 reverse	5’ GCACTTGCGAGATCCTTCTCA 3’
Ms-Miro1 forward	5’ GCAGAATTCCCTGCTTGATTG 3’
Ms-Miro1 reverse	5’ ATGTTCCTGTTTGACTTCATGCA 3’
Ms-Mitofusion 1 forward	5’ GCAACCGAGAAGCTGCAGAT 3’
Ms-Mitofusion 1 reverse	5’ CTGTACTTGGTGGCTGCAGTTT 3’
Ms-OPA1 forward	5’ CCTTCAGCGATAACGTGGAT 3’
Ms-OPA1 reverse	5’ CCGGGAACCACGTTTGC 3’
Ms-Mito fusion 2 forward	5’ CAGTCCGGGCCAAGCA 3’
Ms-Mito fusion 2 reverse	5’ GTGCAGGGAATCCATGATGAG 3’
Ms-DRP1 forward	5’ GCGCTGATCCCGGTCAT 3’
Ms-DRP1 reverse	5’ CCGCACCCACTGTGTTGA 3’
Ms-Actin forward	5’ TCACGGAGGCACGTTGCT 3’
Ms-Actin reverse	5’ CCGAGTGCACGTTCTCCAT 3’

Supplementary Table 2 List of primers for mtDNA contents

Ms-Dloop1 Forward	5’ AATCTACCATCCTCCGTGAAACC 3’
Ms-Dloop1 Reverse	5’ TCAGTTTAGCTACCCCCAAGTTTAA 3’
Ms-COX1 Forward	5’ TGCTCCTGATATAGCATTCCCACGA 3’
Ms-COX1 Reverse	5’ TCCACCATGAGCAATTCCAGCGG 3’
Ms-ND4 Forward	5’ AACGGATCCACAGCCGTA 3’
Ms-ND4 Reverse	5’ AGTCCTCGGGCCATGATT 3’
Ms-ND4 Reverse	5’ GGACAAATATCATTTTGAGGAGCTACT 3’
Ms-16S Reverse	5’ GCAAATCCACCTCATAATCATTGA 3’
Ms-RNR-S Forward	5’ CTAGCTCATGTGTCAAGACCCTCTT 3’
Ms-RNR-S Reverse	5’ GCCAGCACGTTTCTCTCGTT 3’
D-COX1 Forward	5’ TGCTCCTGATATAGCATTCCCACGA 3’
D-COX1 Reverse	5’ TCCACCATGAGCAATTCCAGCGG 3’
D-CytB Forward	5’ GGACAAATATCATTTTGAGGAGCTACT 3’
D-CytB Reverse	5’ GCAAATCCACCTCATAATCATTGA 3’
D-RPL32 Forward	5’ AGGCCCAAGATCGTGAAGAA 3’
D-RPL 32 Reverse	5’ TGTGCACCAGGAACTTCTTGAA 3’

Supplementary Figure legends

Supplementary Figure 1. Data related with mitochondrial functions in wild type flies

a) Relative brain ATP level change during aging. The brain ATP level of 15 days-old is set as 1, and the relative ratios of the other ages are shown. 40 fly heads were collected for each experiment. Trial N=3. One-way ANOVA with Tukey's post hoc test. * P=0.0319, * P= 0.0047. **b)** The mtDNA contents change during aging. mtDNA contents is determined by the ratio of mtDNA/nuclear DNA (Rpl32). The level of 1 day-old flies is set as 1, and the relative ratios of the other ages are shown. 40 fly heads were collected for each experiment. Trial N=3. One-way ANOVA with Tukey's post hoc test. **** P<0.0001, ** P=0.0015. **c)** Cytosolic calcium concentration ($[Ca^{2+}]_{cyto.}$) in brain neurons during aging. The ratio of 340nm/380nm of fura-2AM is quantified to indicate the relative cytosolic calcium level $[Ca^{2+}]_{cyto.}$. Trial N=24, 6, 9, 15, 9, 18, 15 and 6 (from the left to right). One-way ANOVA with Tukey's post hoc test. ** P= 0.0052, * P=0.0207. **d)** Vimar protein level change during aging in w^{1118} flies. Uncropped blots are shown in Supplementary Figure 9. Each sample collected 50 fly heads. Sample of 1 day-old is set as 1. Trial N=3. One-way ANOVA with Tukey's post hoc test. *** P=0.0008. **e)** qRT-PCR to quantify the transcripts of mitochondrial regulatory genes during brain aging in *CS* flies. Vimar, Miro, DRP1, OPA1, MFN1, and MFN2 mRNA levels (Primer sets are listed in the Supplementary Table 1). Trial N=4. One-way ANOVA with Tukey's post hoc test. *** P=0.0003, NS for P=0.3163, **** P<0.0001, * P=0.034, ** P=0.0098. **f)** qRT-PCR to quantify the transcripts of mitochondrial regulatory genes during brain aging in w^{1118} flies. Vimar, Miro, DRP1, OPA1, MFN1, and MFN2 mRNA levels. Trial N=4. One-way ANOVA with Tukey's post hoc test. **** P<0.0001, **** P<0.0001, * P= 0.0382, * P=0.0197. **g)** qRT-PCR to quantify the transcripts of mitochondrial regulatory of *Elav-GS>Vimar* fed with RU486 (RU+) or not (RU-). DRP1, OPA1, MFN1, and MFN2 mRNA levels. Trial N=3. One-way ANOVA with Tukey's post hoc test. **** P<0.0001, NS for P=0.8907, 0.3512, 0.4118. **h)** qRT-PCR to quantify the transcripts of mitochondrial regulatory of *Elav-GS>Vimar RNAi* fed with RU486 (RU+) or not (RU-). DRP1, OPA1, MFN1, and MFN2 mRNA levels. Trial N=3. Error bars are mean ± SE. One-way ANOVA with Tukey's post hoc test. P=0.999, 0.6724, 0.0949, 0.1255.

Supplementary Figure 1

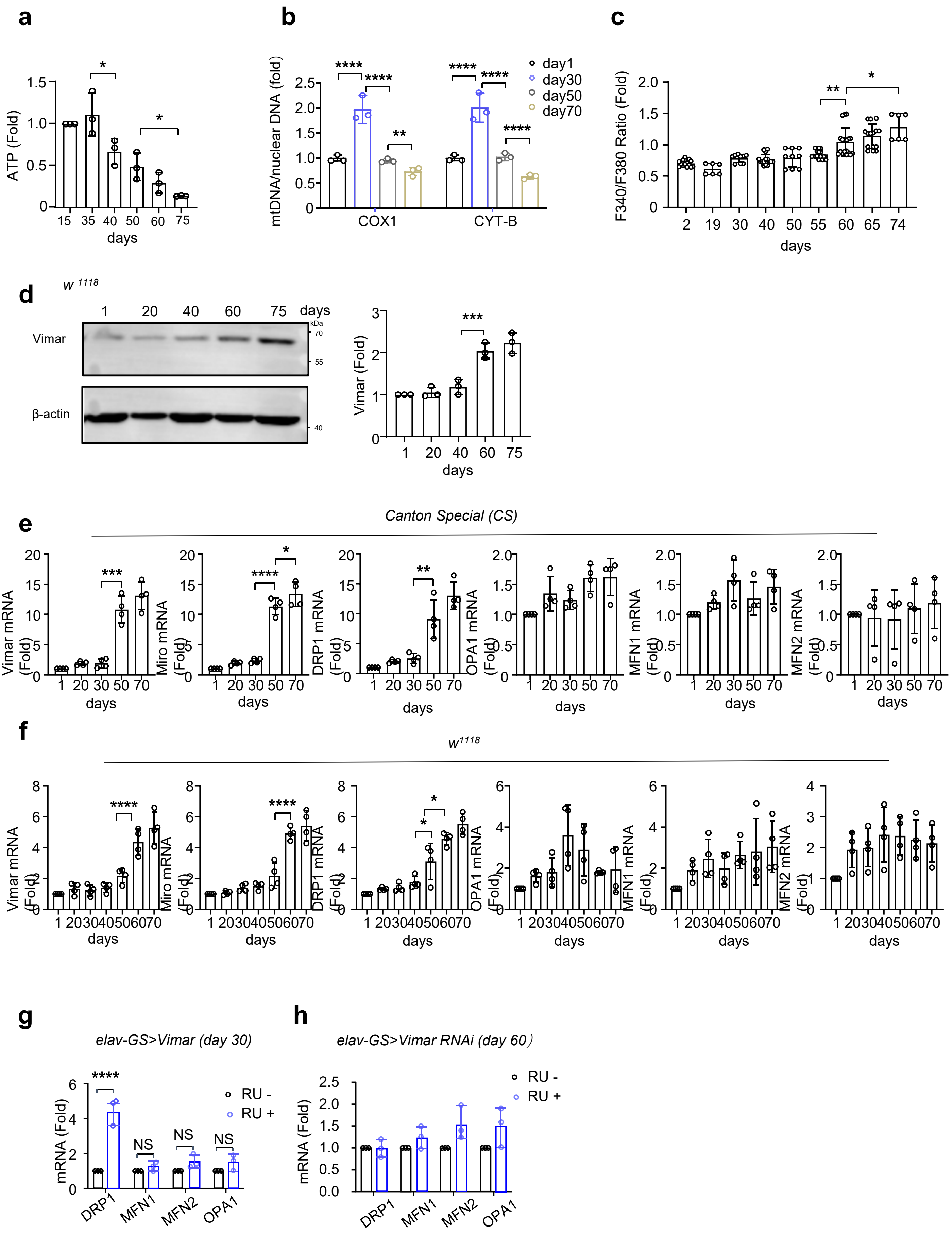

Supplementary Figure 2. Data related with Vimar in brain aging

a) Survival curve of *DA-GS>GFP* fed with different concentration of RU486. RU486 was fed at 1 day-old. Trial N=149 (the grey line, 0 μM RU486); 162 (the purple line, 200 μM RU486); 125 (the blue line, 500 μM RU486). Median lifespan: 0 μM = 56 days; 200 μM = 58 days; 500 μM =58 days. Long-rank test, P=0.484,0.458. **b**) Representative Western bolt of Vimar. Uncropped blots are shown in Supplementary Figure 10. *elav-GS>Vimar* (on 2nd) flies were fed with RU486 (500μM, RU+) or not (RU-). β-actin (the upper band) is shown as the protein loading control from the same blot. The band intensity of RU- brain sample is set as 1, and the relative ratios of the other ages are shown. Trial N=3. Mann–Whitney t-test. ** P=0.003. **c**) Representative Western bolt of Vimar. *DA-GS>Vimar* (on 3rd) flies were fed with RU486 (500 μM, RU+) or not (RU-). β-actin (the upper band) is shown as the protein loading control from the same blot. Uncropped blots are shown in Supplementary Figure 10. The band intensity of RU- brain sample is set as 1, and the relative ratios of the other ages are shown. Trial N=3. Mann–Whitney t-test. ** P=0.0041. **d)** Survival curve of *DA-GS>Vimar* fed with RU486 (500 μM,RU+) or not (RU-), RU486 was fed from 1 day-old. N=133 (the gray line, RU-), N=177 (the purple line RU+). Median lifespan: RU- = 53 days; RU+ = 47 days. Long-rank test, P<0.0001. **e**) Climbing assay. *DA-GS>Vimar* (on 3rd) flies were fed with RU486 (500μM, RU+) or not (RU-). 40 days-old flies were tested with 20 flies tested for each trial. Trial N=10. Mann–Whitney t-test. ***P=0.0002. **f**) Representative live image of mitochondrial morphology stained by Mito Tracker green in brain neurons. The genotype is *DA-GS-Gal4>Vimar* (on 3rd). The flies were 40 days-old. Scale bar, 5 μm. The mitochondrial size of control (RU-) was set as 1, and the relative ratio of Vimar overexpression (RU+) is shown. Trial N=6. Mann–Whitney t-test. *** P=0.0004. **g)** TUNEL assay to determine apoptosis in Vimar overexpression flies. Brain slides were immuostained with the TUNEL staining kit. Representative images were shown for each condition indicated on the micrograph. Scar bar=10 μm. Trail N=3 for each condition. **h)** Effect of Vimar overexpression (*elav-GS>Vimar*) on the daily energy expenditure by measured CO_2 production. Each experiment tested 3-5 flies. Trial N=5. Unpaired t-test. **** P<0.0001. **i)** The ATP level was normalized to protein concentration first. The same genotype as in **h**. The average ATP level of control (RU-) is set as 1, and the relative ratio of the Vimar overexpression (RU+) are shown. Every experiment collected 40 fly heads. Trial N=3. Unpaired t-test. **** P<0.0001. **j**) Effect of Vimar overexpression (*elav-GS>Vimar* (on 2nd)) on citrate synthase (CS) activity. Each experiment collected 40 fly heads. The CS activity was normalized to protein concentration first. The average CS activity of control (RU-) was set as 1, and the relative ratios of the Vimar overexpression (RU+) are shown. Trial N=4. Unpaired t-test. **** P<0.0001. **k**) Representative live image of mitochondrial membrane potential stained by TMRM. The genotype is *elav-GS-Gal4>Vimar* (on 2nd). The flies were 40 days-old. Scale bar, 10 μm. The fluorescent intensity of control (RU-) was set as 1, and the relative ratio of Vimar overexpression (RU+) is shown. Trial N=7. Unpaired t-test. ****P<0.0001. **l**) Brain cytosolic calcium concentration ($[Ca^{2+}]_{cyto.}$) measured by Fura-2. Wild type control is w^{1118}; calcium overload fly is *hs-GluR1*^{Lc}. Each point is the averaged intensity of 3 repeated

measurements. Trial N=4. Unpaired t-test. **P=0.0049. **m**) Effect of Vimar overexpression under *hs-GluR1*^{Lc} background (*DA-GS-Gal4>Vimar* (on 3rd); *hs-GluR1*^{Lc}) on the brain ATP level. The flies were 40 days-old. The ATP level of control (RU-) was set as 1, and the relative ratio of Vimar overexpression (RU+) to the control (RU-) is shown. Trial N=3. One-way ANOVA with Tukey's post hoc test. ***P=0.0003, ***P=0.0003. **n**) Brain CS activity. The CS activity were normalized to protein concentration first. The same genotype as in **m**. Every experiment collected 40 fly heads. Trial N=4. Error bars are mean ± SE. One-way ANOVA with Tukey's post hoc test. **P=0.0037, **P=0.0024. **o)** Survival curve on paraquat induced oxidative stress. Paraquat (5 mM) was fed to the flies same as **m**. Compared with control group, 120 flies were tested for every group. long-rank(mantel-Cox) test, P<0.0001 (compared grey line to purple line), P<0.0001 (compared grey line to red line), P=0.9565 (compared red line to purple line).

Supplementary Figure 2

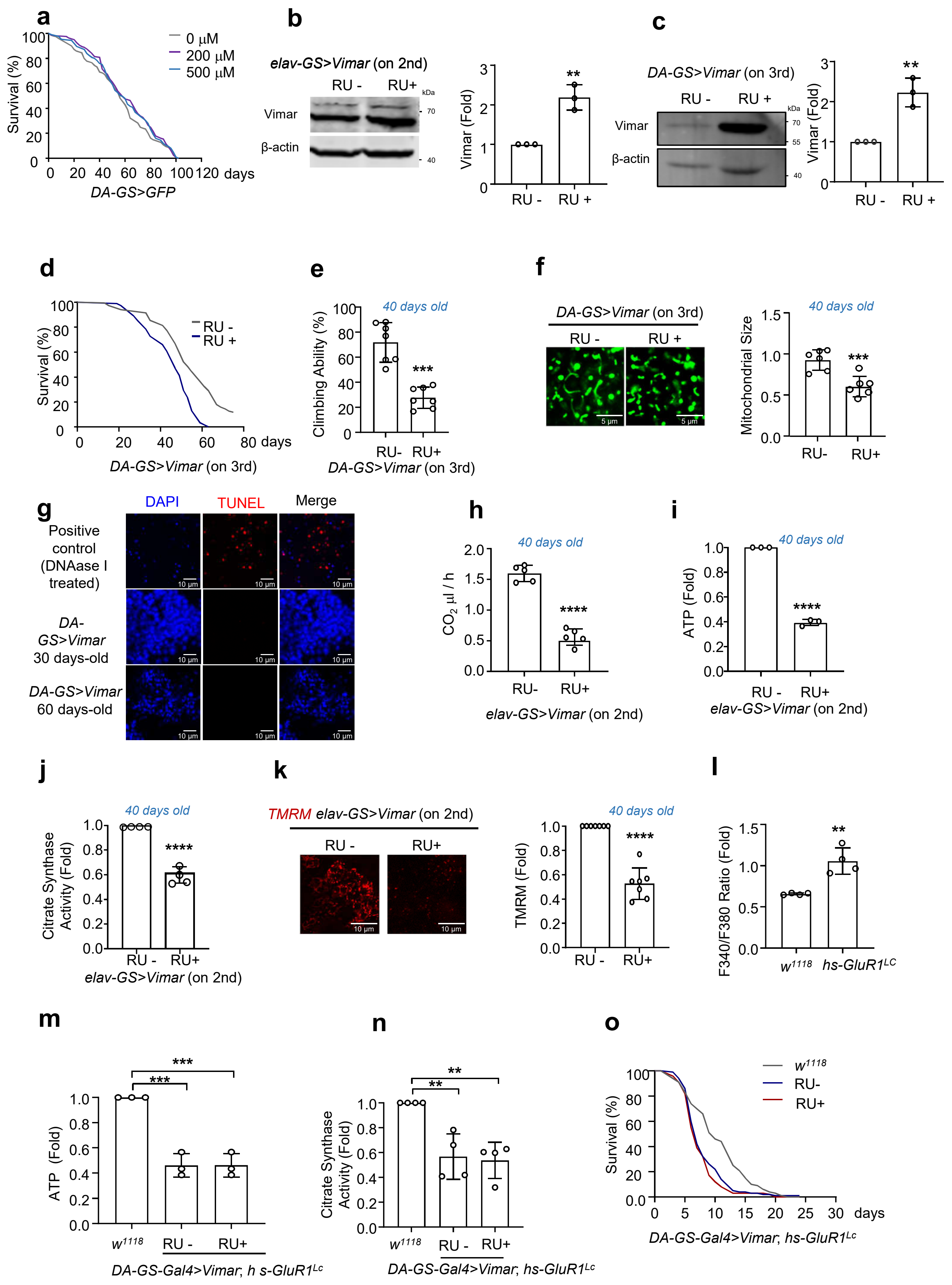

Supplementary Figure 3. Data related with Vimar RNAi effect on aging

a) Representative Western bolt of Vimar in the *elav-GS>Vimar RNAi* (Vimar RNAi transgene is on the 3rd chromosome). Uncropped blots are shown in Supplementary Figure 11. Fed with RU486 (500 μM) or not, Vimar protein level is shown. β-actin from the same blot is used as the protein loading control. The value of RU- as 1, and the ratio of RU+ is shown. Trial N=3. Unpaired t-test. ***P=0.0004. **b**) Climbing assay. The same fly group as **a** is tested, with 55 days-old of flies. Each experiment tested 20 flies. Trial N=7 (RU-), 11 (RU+ all-life) and 10 (RU+ half-life). One-way ANOVA with Tukey's post hoc test. NS for P=0.213, *P=0.0222. **c)** Daily energy expenditure measured by CO_2 production. The same fly group as **a** is tested, with 55 days-old of flies. Each experiment tested 3-5 flies, Trial N=5. One-way ANOVA with Tukey's post hoc test. *P=0.0262, **P=0.0023. **d**) Representative live image of mitochondrial membrane potential stained by TMRM. The genotype is *elav-GS-Gal4>Vimar RNAi* (the Vimar RNAi transgene is on the 3rd chromosome). The flies were 55 days old. Vimar RNAi expression from 1 day-old (RU+ all) or from 30 days-old (RU+ late) are shown. Scale bar, 5 μm. The TMRM intensity of the control (RU-) is set as 1, and the relative ratio of RU486+ are shown. Trial N=4. One-way ANOVA with Tukey's post hoc test. P<0.0001, P<0.0001. **e**) qRT-PCR result of Miro transcript level. The genotype tested are: *DA-GS>Vimar/Miro RNAi* or its background *DA-GS>Vimar/attp2* fed with RU486 or not. The Miro expression level of *DA-GS-gal4>Vimar/attp2* (RU-) is set as 1, and the relative ratios of other fly lines are shown. Trial N=3. One-way ANOVA with Tukey's post hoc test. P<0.0001, P<0.0001. **f)** qRT-PCR result of Vimar transcript level. The same genotype lines as **e**. The Vimar expression level of *DA-GS-gal4>Vimar/attp2* (RU-) is set as 1, and the relative ratios of other fly lines are shown. Trial N=3. Error bars are mean ± SE. One-way ANOVA with Tukey's post hoc test. P<0.0001,P=0.7535, P<0.0001. **g**) Survival curve. Miro down regulation under the background of Vimar overexpression. *DA-GS-Gal4>Vimar/Miro RNAi* fed RU486 (*Miro RNAi* RU+), the red line; or not fed RU486 (*Miro RNAi* RU-), the black line. The genetic background matched control (*attp2*), *DA-GS-Gal4>Vimar/attp2* fed RU486 (attp2 RU+), the green line. Or not fed RU486 (*attp2* RU-), the gray line. Each vial contained 10 female and 10 male flies. The RU486 was given from 1 day-old. Fly N=139 (grey line, *attp2 RU-*); 166 (green line, *attp2* RU+), 177 (black line, *Miro RNAi* RU-), 177 (red line, *Miro RNAi* RU+). Median lifespan: attp2 RU- = 65 days; attp2 RU+ = 48 days; Miro RNAi RU- = 62 days; Miro RNAi RU+ = 69 days. Compared *Miro RNAi* RU+ with *appt2* RU+, Long-rank test, P<0.0001.

Supplementary Figure 3

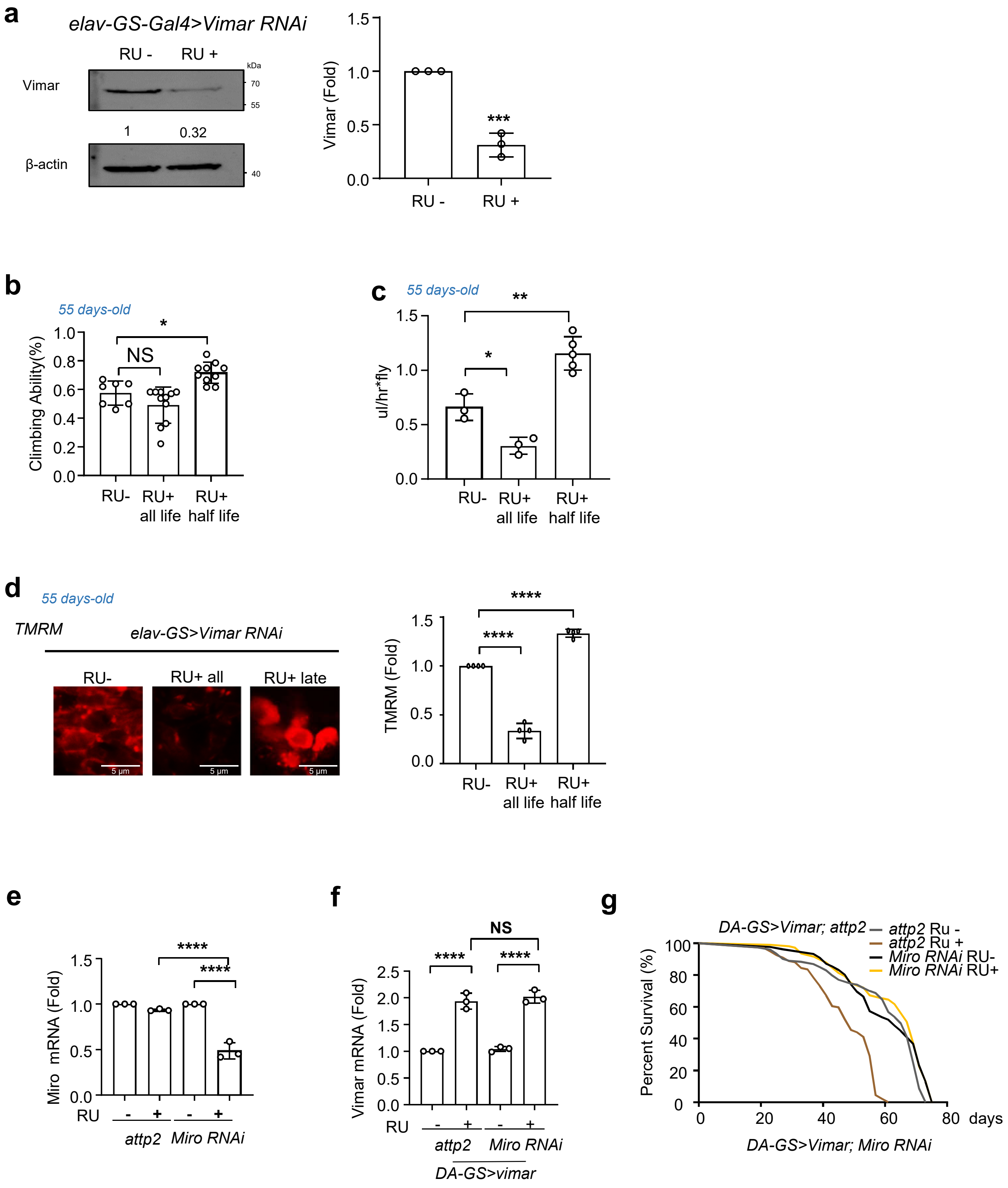

Supplementary Figure 4. Data related with RAPGDS1 function in cultured cells

a) Representative Western blot of cells transfected with *RAP1GDS1* siRNA. β-actin is the protein loading control in the same blot. Uncropped blots are shown in Supplementary Figure 12. **b**) Representative live image mitochondrial morphology changes with time after ionomycin treatment. Scale bar, 5 μm. Mitochondrial length was quantified by image J, with "fragment" (mitochondrial size <0.2 μm^2), "short" (0.2 μm^2 <mitochondrial size <2 μm^2) and long (mitochondrial size >2 μm^2). Control and *RAP1GDS1* RNAi cells, Trial N=6. **c**) Representative live image of mitochondrial morphology in U87-MG cells with *RAP1GDS1* knockdown. Scar bar, 5μm. In **c-g** studies, the SH-SKN cells were used. **d**) Representative live image of ROS level stained by CellROX in cells transfected with a Pljm1-RAP1GDS1-eGFP vector which expresses RAP1GDS1 and eGFP independently. Control was cells transfected with the vector expressed the eGFP alone. *RAP1GDS1* overexpressed cells are also eGFP positive. Scale bar, 20 μm. The red intensity of cells was quantified by ImageJ. Cell number N=5. Unpaired t-test. **** P<0.0001. **e)** Representative live image of mitochondrial morphology in cells transfected with a Pljm1-RAP1GDS1-eGFP vector. Control was cells transfected with the vector expressed the eGFP alone. *RAP1GDS1* overexpressed cells are also eGFP positive (RAP1GDS1, the lower panel). Scale bar, 5μm. Cell number N=8. Unpaired t-test. ** P=0.0024. **f**) Representative live image of $[Ca^{2+}]_{mito.}$ stained by Rhod-2 AM. eGFP positive signal represents cell overexpressed *RAP1GDS1*; eGFP negative signal indicates the control without *RAP1GDS1* overexpression. Scale bar, 10 μm. The florescent intensity of Rhod2-AM was quantified by ImageJ. Cell number N=8 (control); (*RAP1GDS1* overexpression). Unpaired t-test. **** P<0.0001. **g)** Representative live image of mitochondrial membrane potential stained by TMRM. Scale bar, 5 μm. Cell number N=8. Error bars are mean ± SE. Unpaired t-test. **** P<0.0001.

Supplementary Figure 4

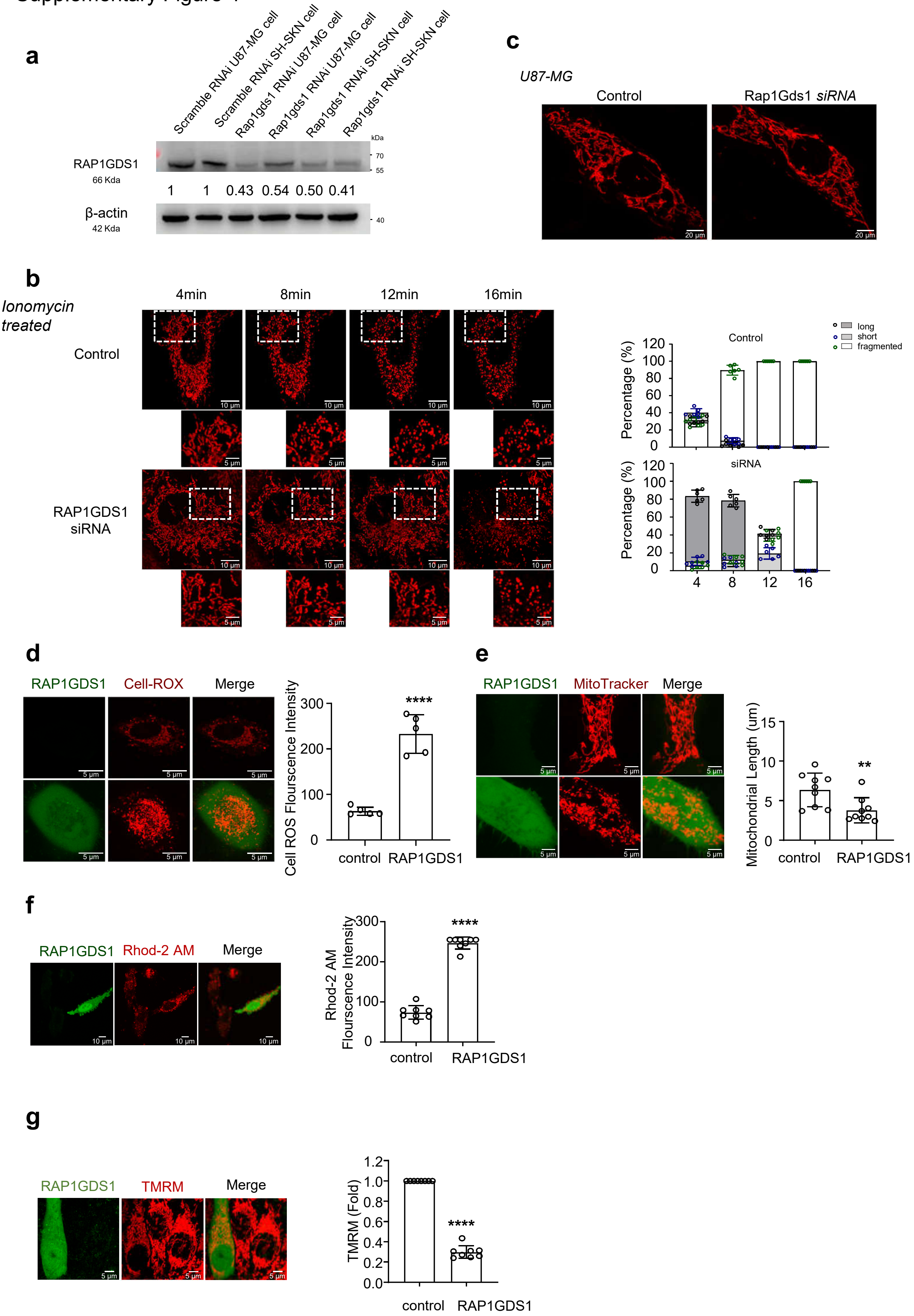

Supplementary Figure 5. Data related with RAP1GDS1/Miro1 effect on mitochondria in mouse brain aging

a) qRT-PCR to quantify the transcripts of mitochondrial regulatory genes during brain aging in mice (Primer sets are listed in the Supplementary Table 1). *RAP1GDS1, Miro-1, MFN1, MFN2, OPA1* and *Drp1* mRNA levels. Trial N=3. One-way ANOVA with Tukey's post hoc test. ****P<0.0001, ****P<0.0001. **b)** The protein level of DRP1, Caspase 3 and cleaved Caspase 3 druing barin aging. Uncropped blots are shown in Supplementary Figure 13. The cleaved caspase 3 protein was not detected in these samples. Trial N=4. One-way ANOVA with Tukey's post hoc test. NS P= 0.9874. **c**) The protein level of RAP1GDS1 and Miro1 expression between young (2 months-old, Young) and old (28 months-old, Old) mice brain. Uncropped blots are shown in Supplementary Figure 13. Mitochondria were extracted from the cortex. VDAC-1 is used as a mitochondrial marker. Trial N=2. **d**) Representative image of mitochondrial morphology of neuronal axons marked by inject *AAV-BBB-Mito DsRed*. Scar bar, 5 μm (for all these images). Mitochondrial morphology was classified as "fragment" (mitochondrial size <0.2 μm^2), "short" (0.2 μm^2 <mitochondrial size <2 μm^2) and long (mitochondrial size >2 μm^2) and quantified by Image J. Cell number, N=6. One-way ANOVA test. ***P=0.0003. **e)** Brain ATP level tissue change during aging. the ATP level was normalized to protein concentration first. The relative ATP level of 2 months-old is set as 1, and the ratios of other age groups are shown. Trial N=5. One-way ANOVA with Tukey's post hoc test. **** P<0.0001. **f)** Brain citrate synthase activity (CS) level tissue change during aging. the CS level was normalized to protein concentration first. The relative CS level of 2 months-old is set as 1, and the ratios of other age groups are shown. Trial N=5. One-way ANOVA with Tukey's post hoc test. * P=0.0299. **g**) qRT-PCR to quantify the mtDNA fold change during aging in mouse brain. mtDNA contents is determined by the ratio of mtDNA/nuclear DNA (Primer sets are listed in the Supplementary Table 2). The level of 2 months-old mice is set as 1, and the relative ratios of the other ages are shown. Trial N=3. Error bars are mean ± SE. One-way ANOVA with Tukey's post hoc test. ***P=0.0001, NS 0.9063, *** 0.0001, * 0.0233, *** 0.0001, * 0.0323, *** 0.0001, NS 0.9949, *** 0.0001, ** 0.0073.

Supplementary Figure 5

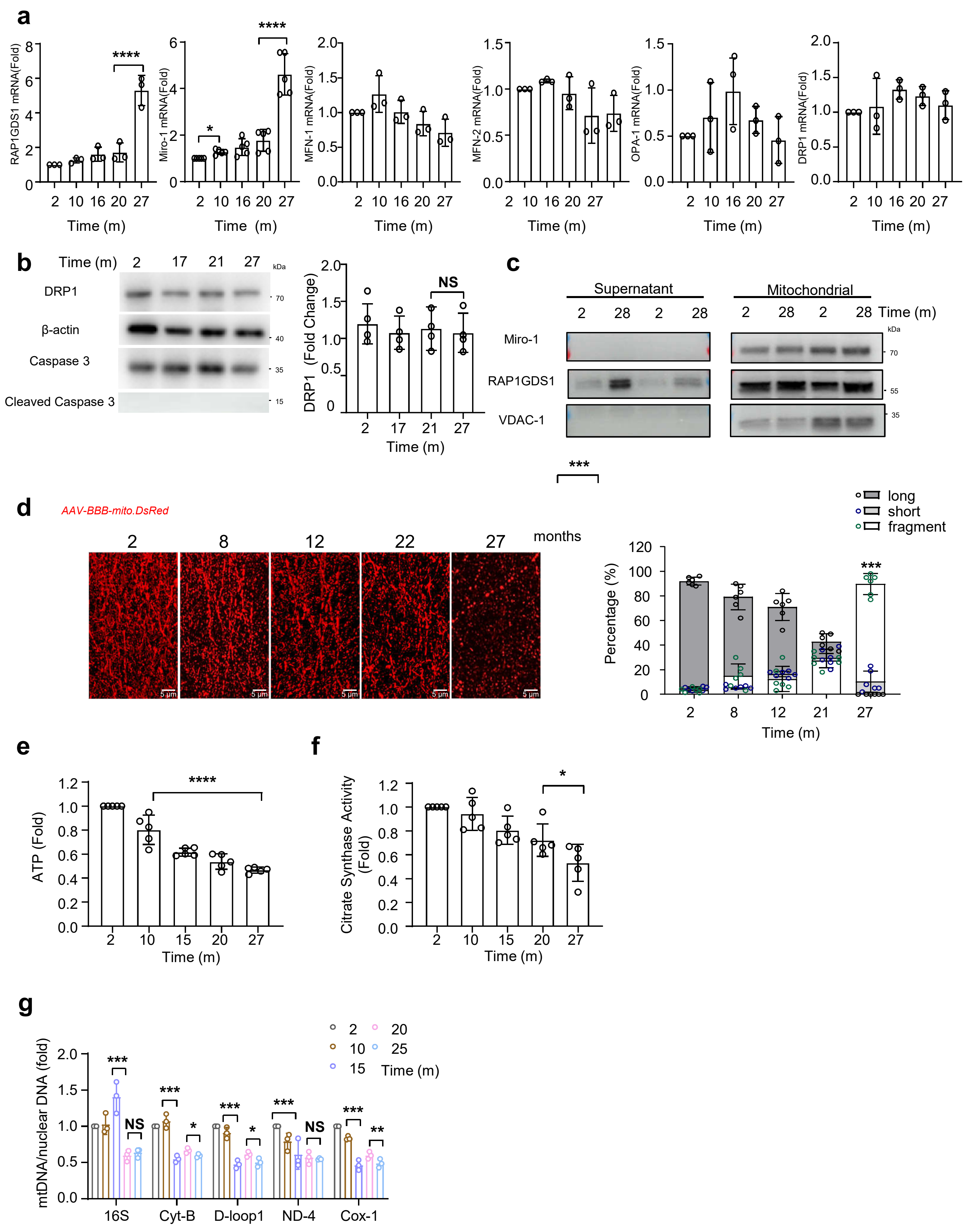

Supplementary Figure 6. Data related with effect of RAP1GDS1 transgene and knockdown on mouse brain aging

a) RAP1GDS1 protein level in the mice with neuron-specific overexpression of *RAP1GDS1*. Uncropped blots are shown in Supplementary Figure14. The mouse genotype is MAP2-Cre-ERT2+/-; *RAP1GDS1*ox/+. Tamoxifen was injected for 7 days when mice were 2 months-old. The mice were sacrificed at 8 months-old. Compared with tamoxifen injection or not, RAP1GDS1 protein levels are shown by Western blot. Quantification is normalized to the protein loading control, β-actin. **b)** Brain ATP level. Control (8 months-old) and *RAP1GDS1* overexpression (8 months-old) are compared. Mice N=5. Unpaired t-test, ** P=0.0086. **c)** Citrate synthase activity. Same samples as **b**. Mice N=5. Unpaired t-test.* P=0.016. **d)** Nest building assay. Mice N=6. One-way ANOVA with Tukey's post hoc test. **** P<0.0001, **** P<0.0001. **e)** Rotarod assay. The maximum duration a mouse could hang on the bar. Mice N=6. One-way ANOVA with Tukey's post hoc test. **** P<0.0001, *** P=0.0002, ** P=0.0026. **f)** The forelimb strength assay. Mice N=6. One-way ANOVA with Tukey's post hoc test. *** P=0.0004, ** P=0.0046, **** P<0.0001. **g**) RAP1GDS1 protein level in the mice with neuron-specific knockout of *RAP1GDS1*. Uncropped blots are shown in Supplementary Figure 14. The mouse genotype is MAP2-Cre-ERT2$^{+/-}$; *RAP1GDS1*$^{ko/+}$. 3 months-old mice were injected with D-galactose daily for 20 days. Then, the mice were injected tamoxifen to heterozygous knockdown of *RAP1GDS1*. These mice were sacrificed at 5 months-old. Compared with tamoxifen injection or not, RAP1GDS1 protein levels are shown by Western blot, and quantified by normalized to β-actin. **h**) Nest building assay. Mice N=6. Unpaired t-test. ** P=0.002. **i)** Rotarod assay. The maximum time of a mouse hanged on the bar (left). From 4 rpm to 40 rpm in 5 minutes, the maximum speed a mouse could hang on the bar(right). Mice N=6. Unpaired t-test. * P=0.026, * P=0.0195. **j**) Novel object recognition assay. Mice N=6. Unpaired t-test. **** P<0.0001. **k**) Immunostaining by VDAC1 to stain mitochondria in the outer molecular layer of prefrontal region. Scale bars, 5 μm. The average mitochondrial size of D-galactose group set as 1, and the relative ratios of D-galactose + KO are shown. Each mouse quantified 10 neurons. Mice N=6. Unpaired t-test. ** P=0.0079. **l)** Brain ATP level. Wild type mice treated with D-galactose, Mice N=6. Heterozygous *RAP1GDS1* knockout mice treated with D-galactose, Mice N=8. Unpaired t-test. ** P=0.0066. **m)** Citrate synthase assay. Wild type mice treated with D-galactose is set as 1. Mice N=8. Error bars are mean ± SE. Unpaired t-test. *** P=0.0007.

Supplementary Figure 6

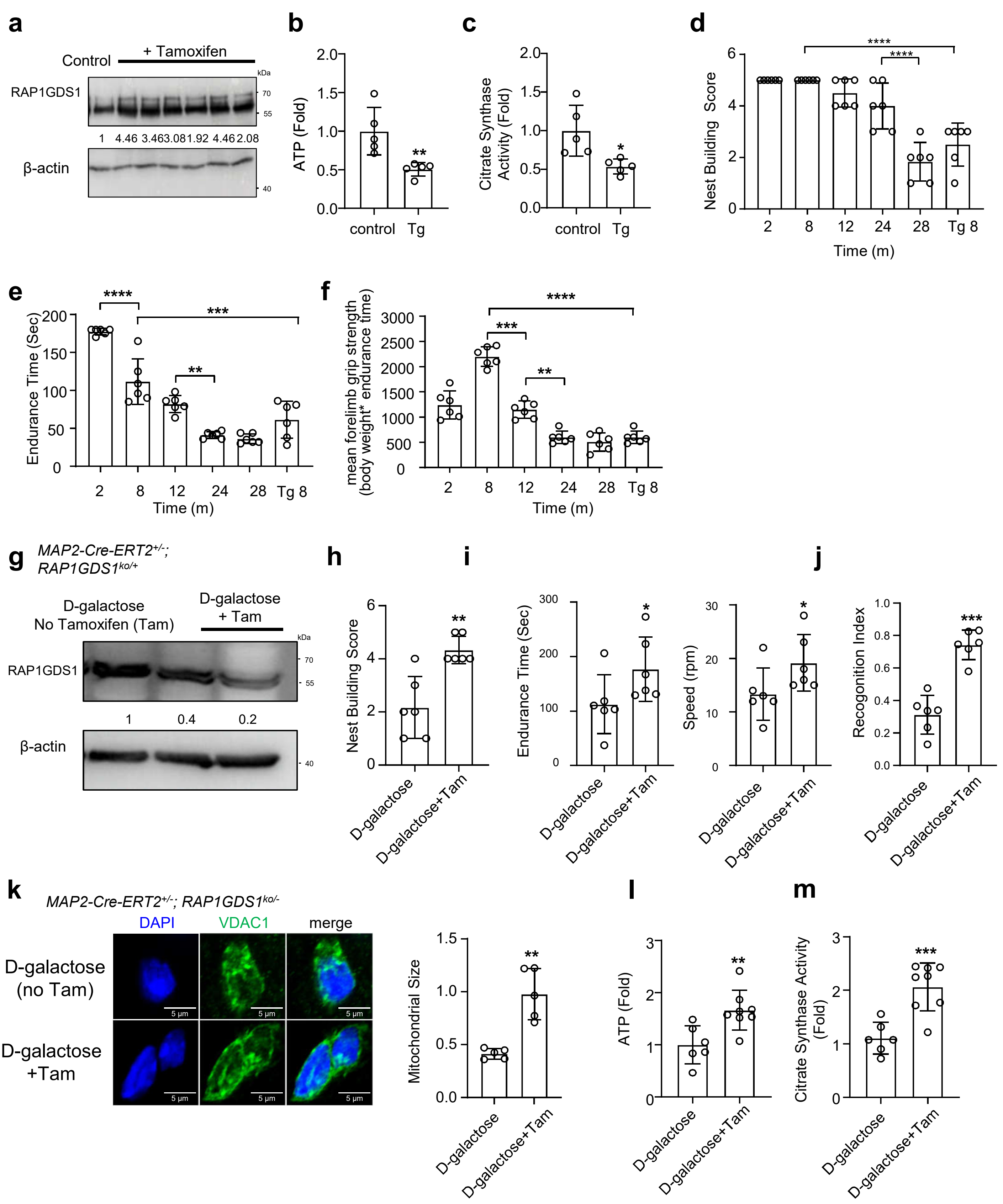

Supplementary Figure 7

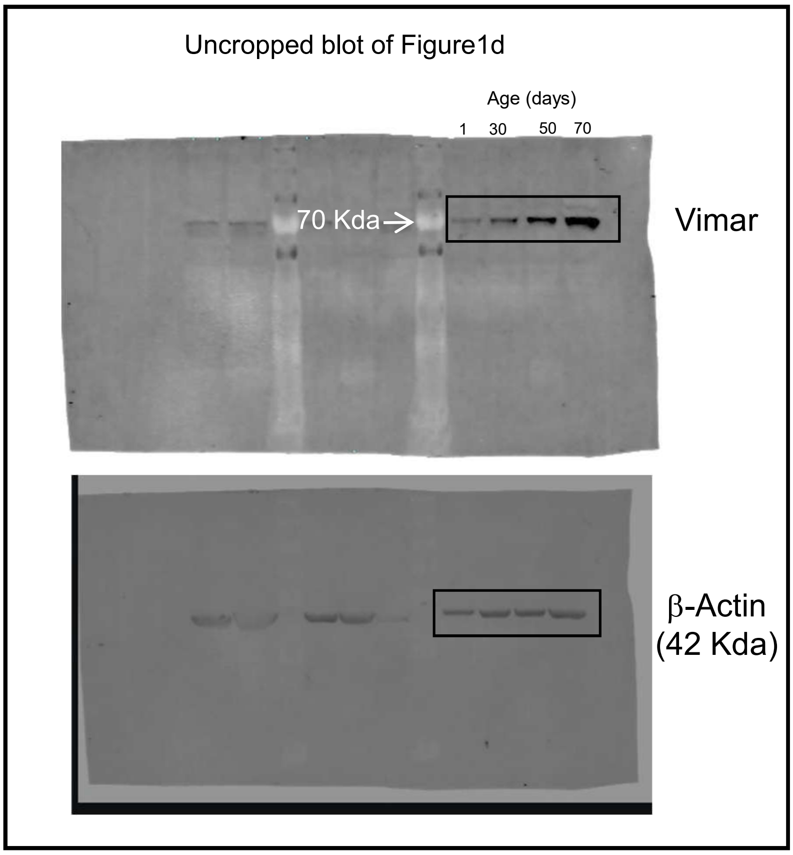

Supplementary Figure 7. Uncropped WB sample of Figure 1d

The protein samples of wild type (*CS*) fly heads were collected with the age of fly listed on the micrograph. For each sample, 50 fly heads were harvested. The size of protein ladder was indicated on the micrograph. The β-actin antibody was from Proteintech (66009-1-Ig).

Note: Primary antibodies were sequentially added to the polyvinylidene fluoride membrane in WB experiments throughout all figures.

Supplementary Figure 8

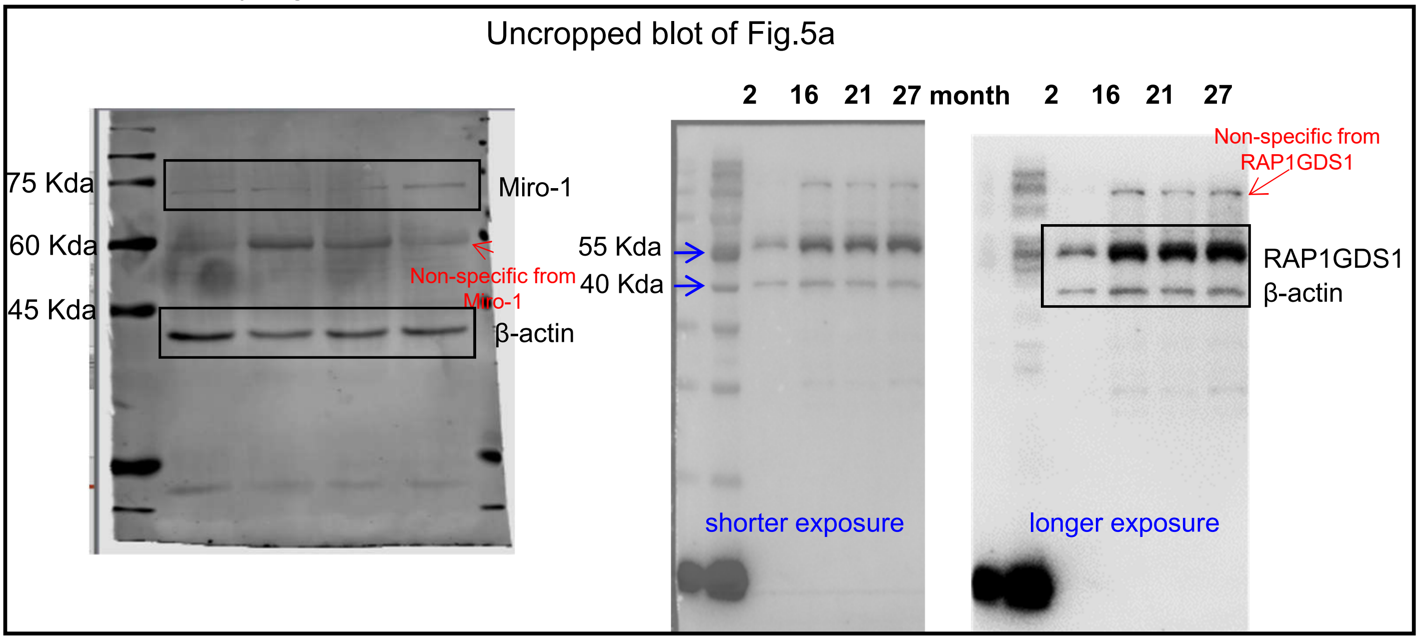

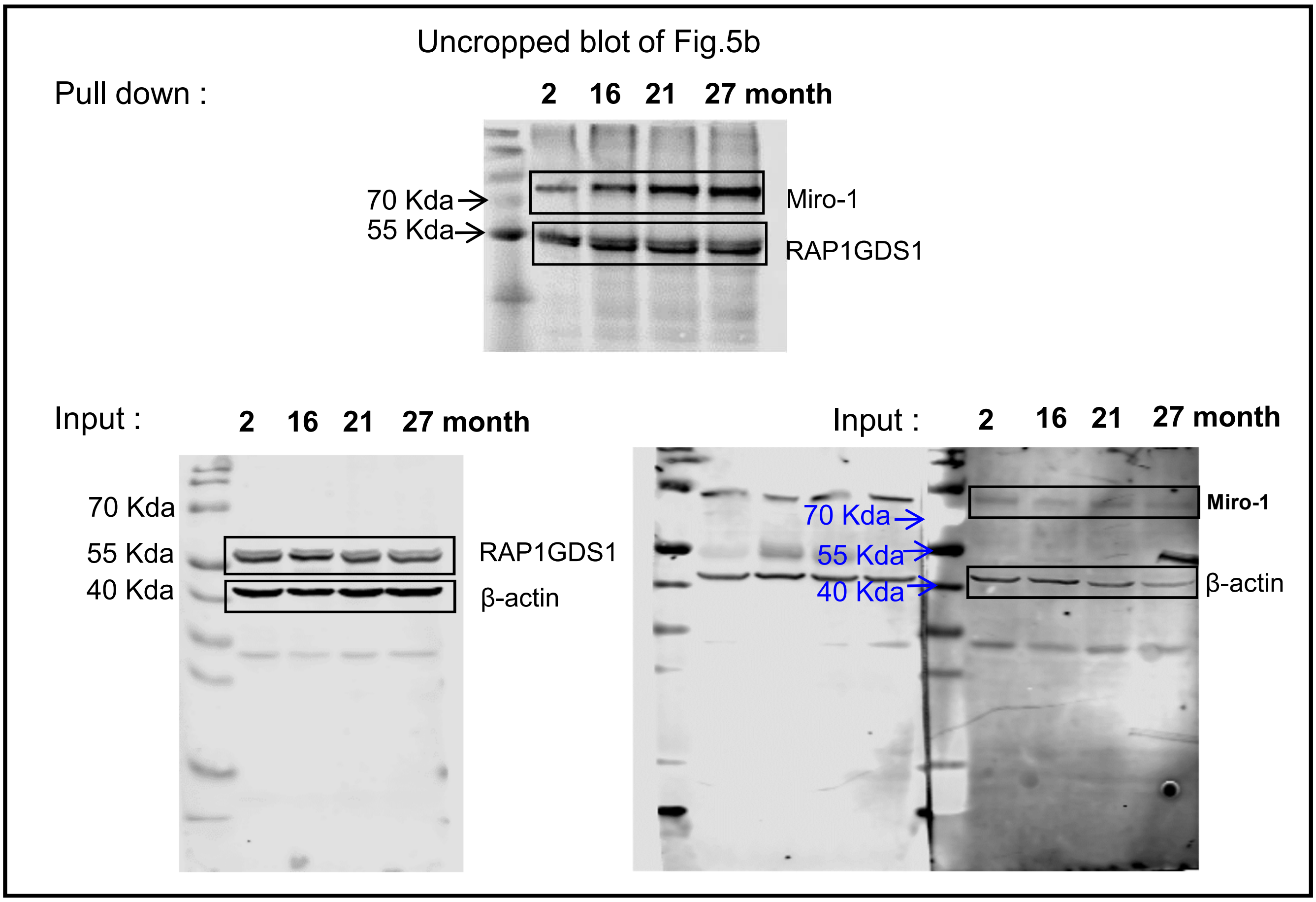

Supplementary Figure 8. Uncropped WB samples of Figure 5a and 5b

The protein samples from wild type mice with different ages listed on the micrograph. Bands of antibody correctly recognized and bands of non-specifically detected were labeled.

Supplementary Figure 9

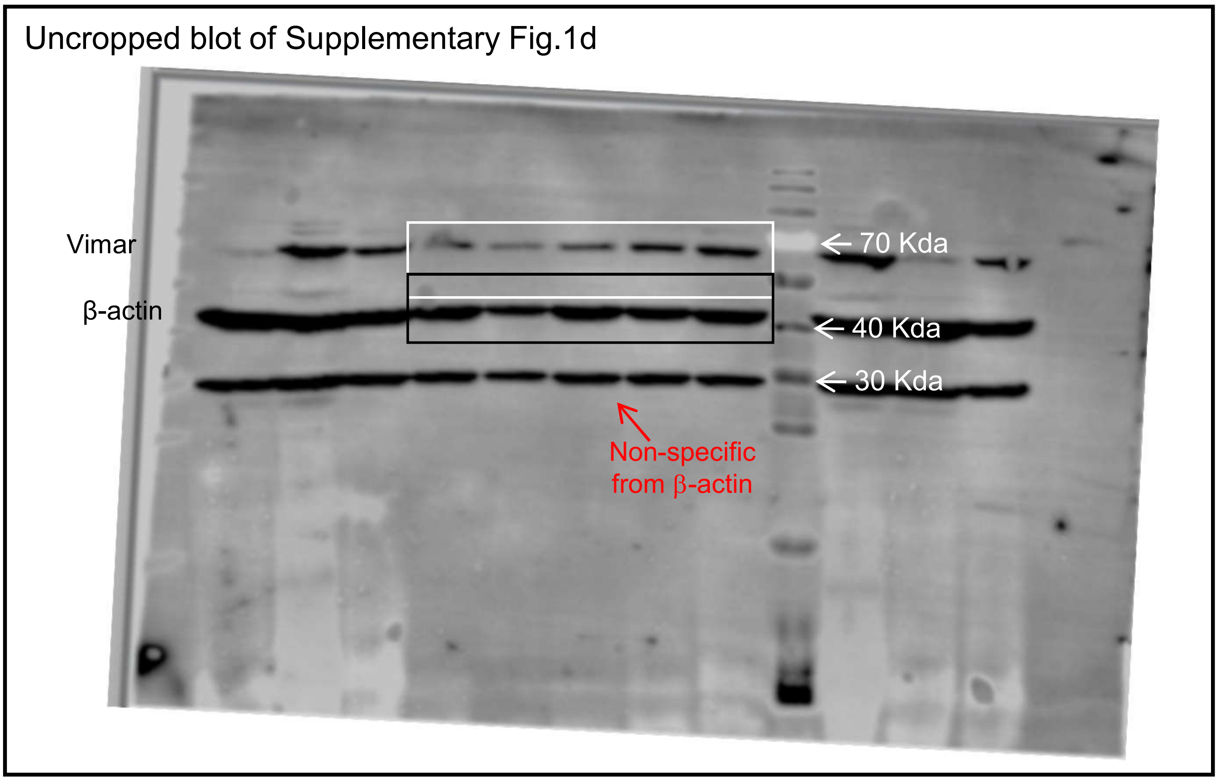

Supplementary Figure 9. Uncropped WB samples of Supplementary Figure 1d

The protein samples from wild type mice with different ages listed on the micrograph. Bands of antibody correctly recognized and bands of non-specifically detected were labeled. The β-actin antibody was from Transgen (HC-201-01). It labels a non-specific band at lease 30 Kda.

Supplementary Figure 10

Uncropped blot of Supplementary Figure 2b

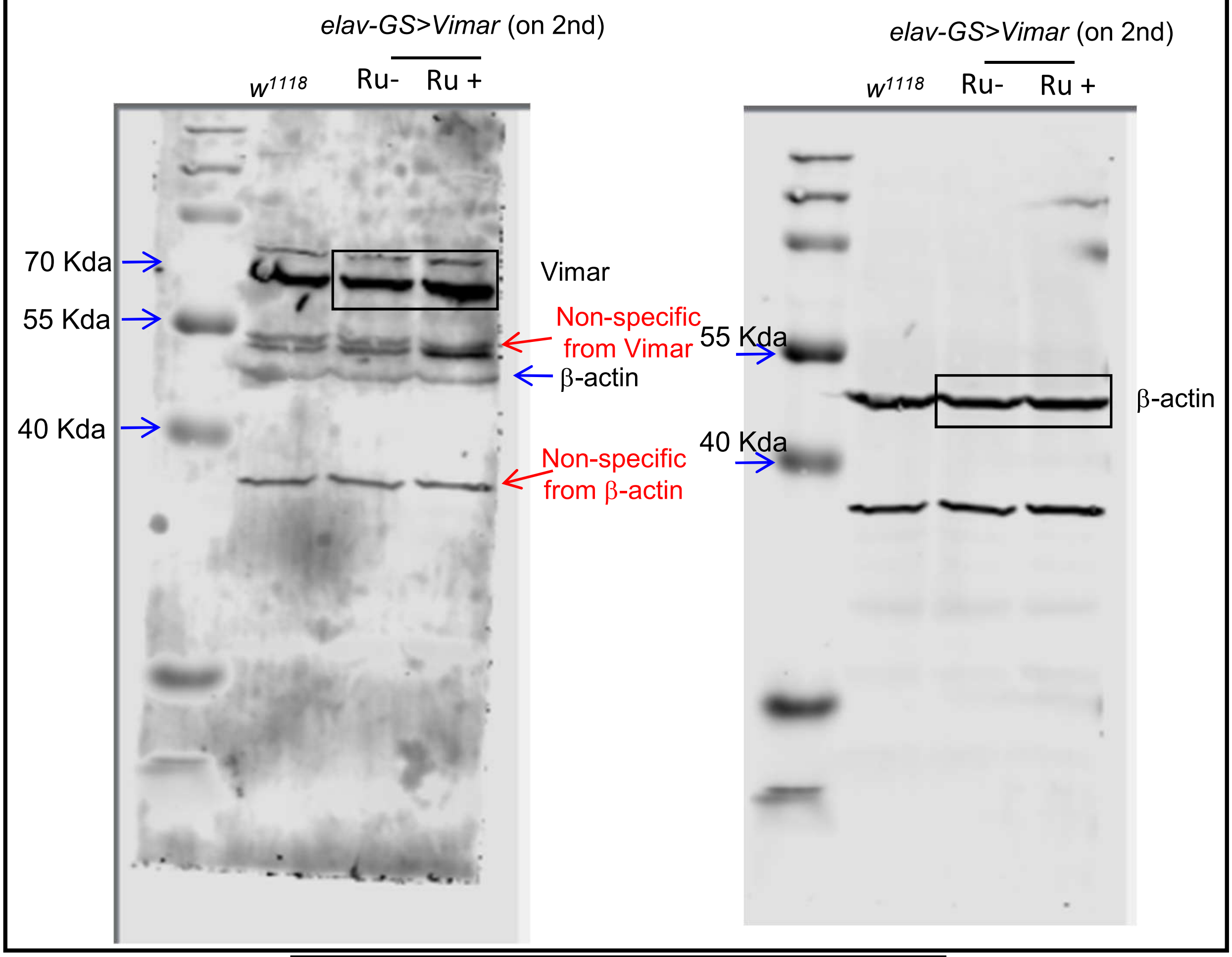

Uncropped blot of Supplementary Figure 2c

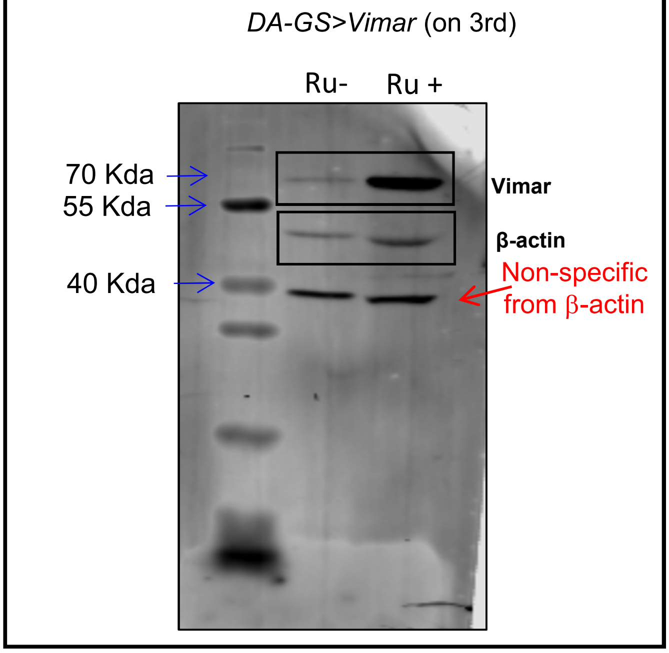

Supplementary Figure 10. Uncropped WB samples of Supplementary Figure 2b and 2c

The protein samples from fly heads with the genotype indicated on the graph. 40 heads were collected for each sample. The β-actin antibody was from Transgen (HC-201-01).

Supplementary Figure 11

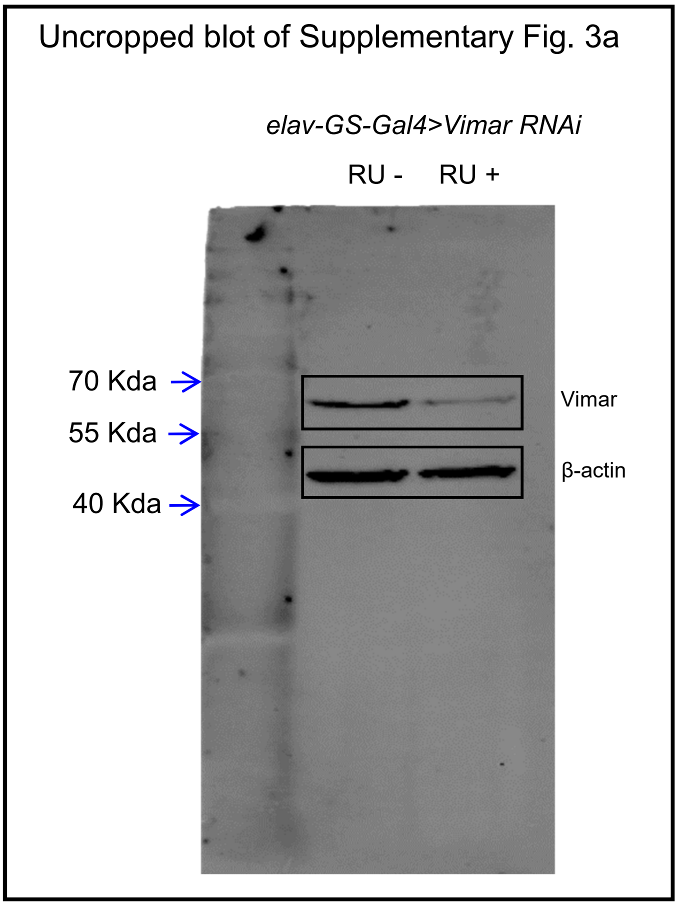

Supplementary Figure 11. Uncropped WB samples of Supplementary Figure 3a

The protein samples from fly heads with the genotype indicated on the graph. 60 heads were collected for each sample. The β-actin antibody was from Proteintech (66009-1-Ig).

Supplementary Figure 12

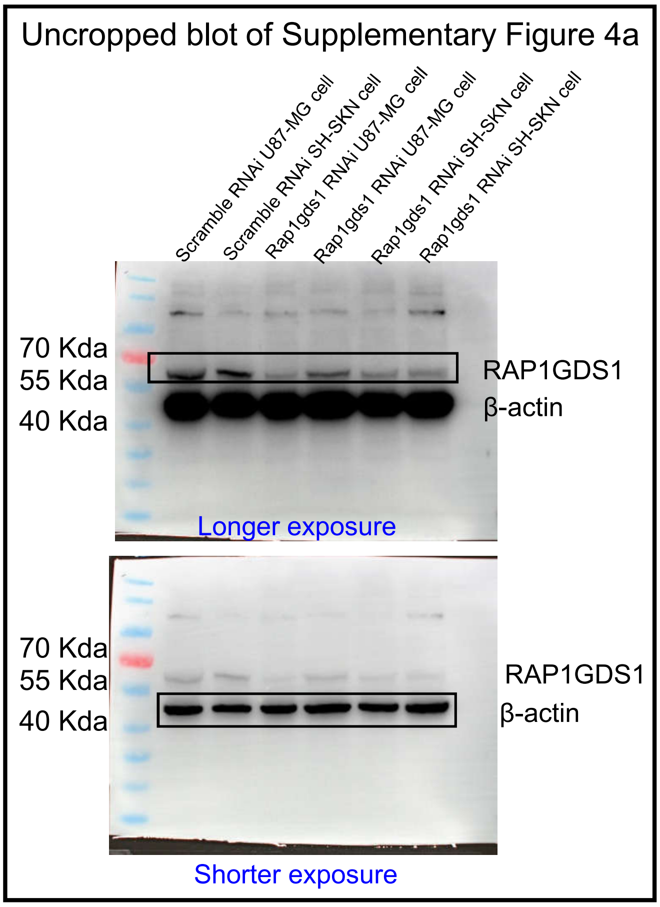

Supplementary Figure 12. Uncropped WB samples of Supplementary Figure 4a

The protein samples from cultured cells with the condition indicated on the graph. The β-actin antibody used were Transgen (HC201).

Supplementary Figure 13

Uncropped blot of Supplementary Figure 5b

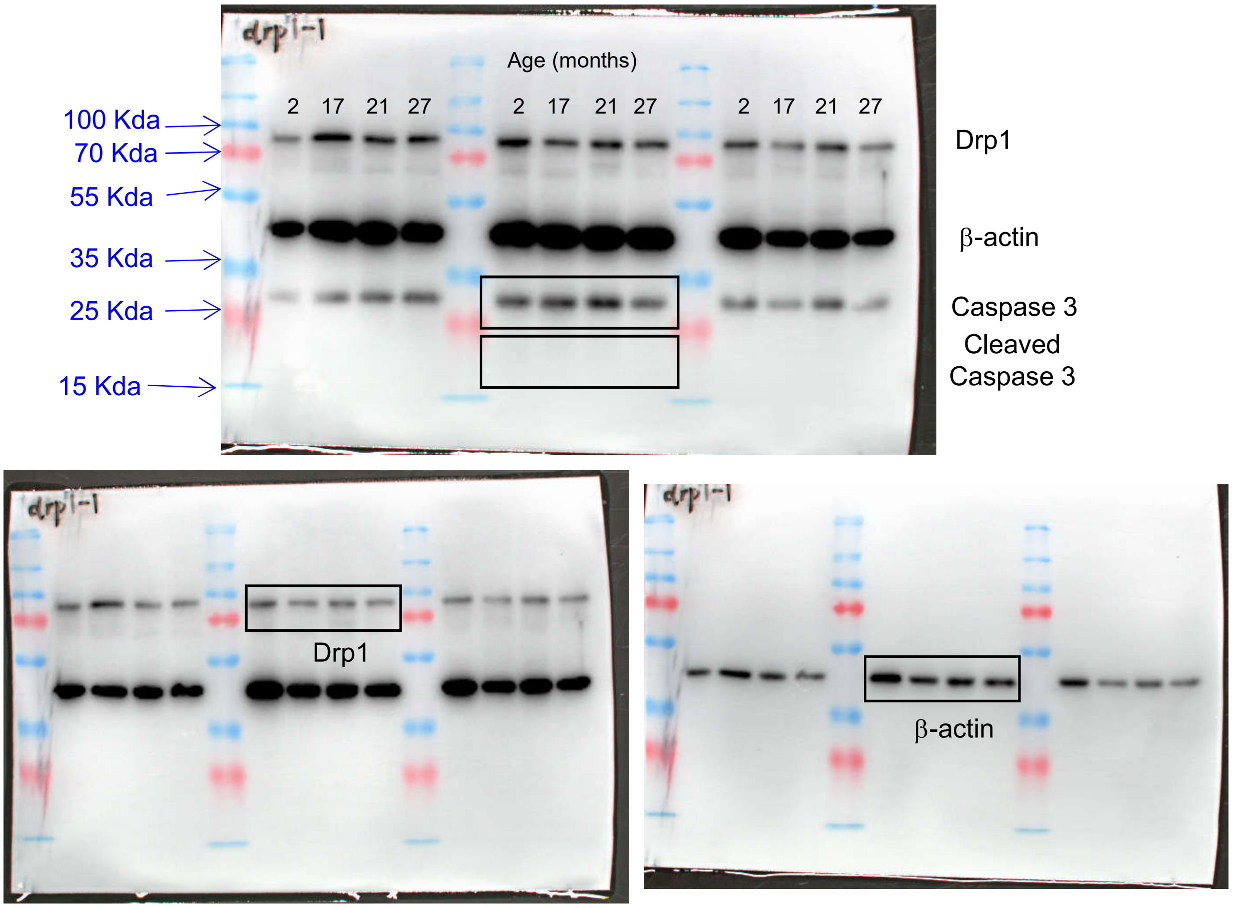

Uncropped blot of Supplementary Figure 5c

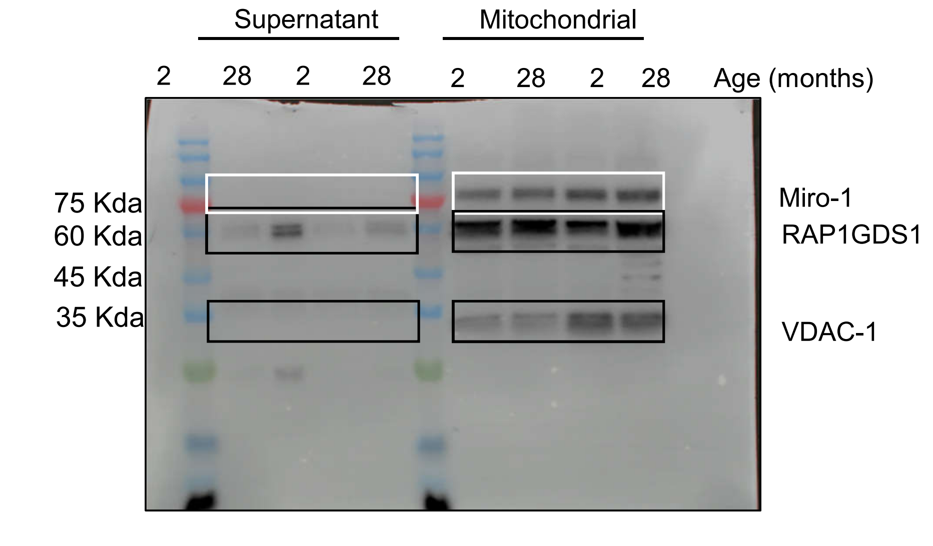

Supplementary Figure 13. Uncropped WB samples of Supplementary Figure 5b and c

5b) The protein samples were from different mice (total 12 mice) with their ages indicated on the micrograph. The three micrographs were from the same polyvinylidene fluoride membrane with different antibody detections. β-actin antibody used were from Transgen (HC201).
5c) The protein samples were rom different mice (total 4 mice) with their ages listed on the micrograph.

Supplementary Figure 14

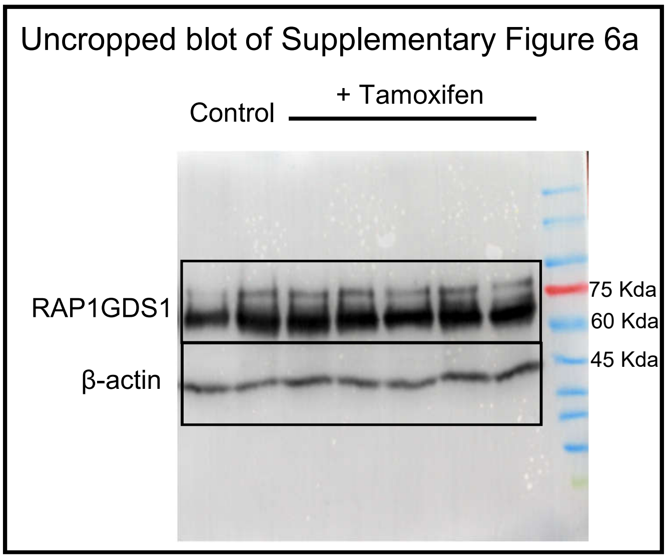

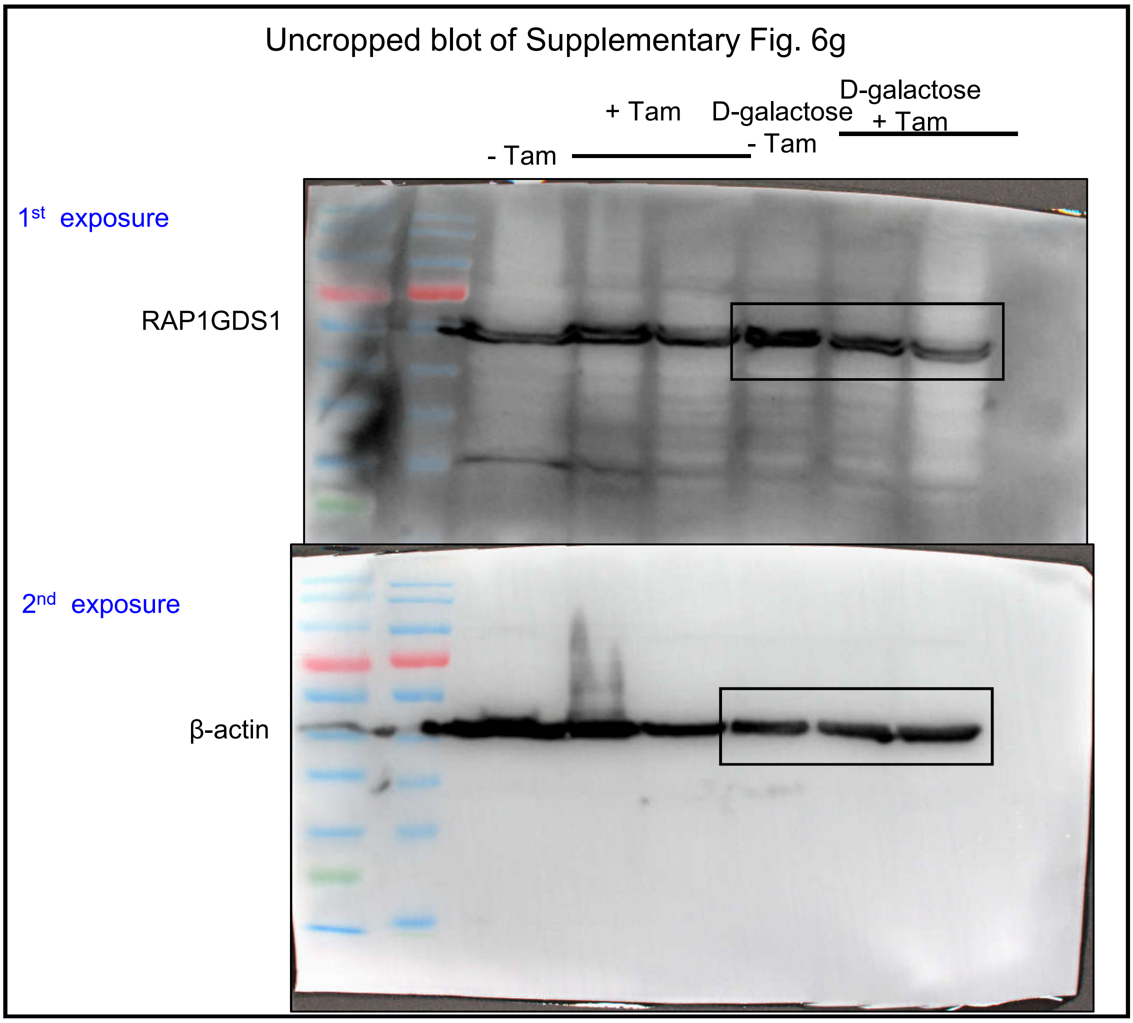

Supplementary Figure 14. Uncropped WB samples of Supplementary Figure 6a and g

The protein samples from different genotype of mice indicated on the graph. The β-actin antibody used were Transgen (HC201).
