# Supplementary material for: Vimar/RAP1GDS1 promotes acceleration of brain aging after flies and mice reach middle age
Source: Commun Biol. 2023 Apr 15;6:420. doi: 10.1038/s42003-023-04822-1 (PMC10105717; doi:10.1038/s42003-023-04822-1)
Supplement: Supplementary file 2 — Description of Additional Supplementary Files [file 42003_2023_4822_MOESM2_ESM.pdf]

Description of Additional Supplementary Files

File name: Supplementary Data 1

Description: Source data underlying figures
